# Positional cloning of quantitative trait nucleotides for blood pressure and cardiac QT-interval by targeted CRISPR/Cas9 editing of a novel long non-coding RNA

**DOI:** 10.1371/journal.pgen.1006961

**Published:** 2017-08-21

**Authors:** Xi Cheng, Harshal Waghulde, Blair Mell, Eric E. Morgan, Shondra M. Pruett-Miller, Bina Joe

**Affiliations:** 1 Program in Physiological Genomics, Center for Hypertension and Personalized Medicine, Department of Physiology and Pharmacology, University of Toledo College of Medicine and Life Sciences, Toledo, OH, United States of America; 2 Department of Radiology, University of Toledo Medical Center, Toledo, OH, United States of America; 3 Department of Cell & Molecular Biology, Center for Advanced Genome Engineering, St. Jude Children’s Research Hospital, Memphis, TN, United States of America; Medical College of Wisconsin, UNITED STATES

## Abstract

Multiple GWAS studies have reported strong association of cardiac QT-interval to a region on HSA17. Interestingly, a rat locus homologous to this region is also linked to QT-intervals. The high resolution positional mapping study located the rat QT-interval locus to a <42.5kb region on RNO10. This region contained no variants in protein-coding sequences, but a prominent contiguous 19bp indel polymorphism was noted within a novel predicted long non-coding RNA (lncRNA), which we named as *Rffl-lnc1*. To assess the candidacy of this novel lncRNA on QT-interval, targeted CRISPR/Cas9 based genome-engineering approaches were applied on the rat strains used to map this locus. Targeted disruption of the rat *Rffl-lnc1 locus* caused aberrant, short QT-intervals and elevated blood pressure. Further, to specifically examine the significance of the 19bp polymorphism within the *Rffl-lnc1* locus, a CRISPR/Cas9 based targeted knock-in rescue model was constructed by inserting the 19bp into the strain which contained the deletion polymorphism. The knock-in alleles successfully rescued the aberrant QT-interval and blood pressure phenotypes. Further studies revealed that the 19bp polymorphism was necessary and sufficient to recapitulate the phenotypic effect of the previously mapped <42.5kb rat locus. To our knowledge, this study is the first demonstration of a combination of both CRISPR/Cas9 based targeted disruption as well as CRISPR/Cas9 based targeted knock-in rescue approaches applied for a mammalian positional cloning study, which defines the quantitative trait nucleotides (QTNs) within a rat long non-coding RNA as being important for the pleiotropic regulation of both cardiac QT-intervals and blood pressure.

## Author summary

Diseases of the cardiovascular system such as essential hypertension do not have a clear cause, but are known to run in families. The inheritance patterns of essential hypertension and other cardiac diseases suggest that they are not due to a single defective gene but instead are caused by multiple genetic defects that are inherited together in a patient. This complex inheritance makes it difficult to pinpoint the underlying defects. Here, we describe a panel of genetically-engineered rats, using which we have discovered a novel gene, which does not code for any protein, as a gene required for maintenance of normal blood pressure. Structural defects within this non-coding RNA cause hypertension and cardiac short-QT interval. Further, by performing genome surgery to correct the gene defect, we demonstrate the precise error in nucleotides that was inherited and caused hypertension and cardiac short-QT interval syndrome.

## Introduction

It is estimated that hypertension affects nearly 75 million Americans (about 1 in every 3 U.S. adults) [1]. Essential hypertension is the most common type of hypertension and remains a major risk factor for cardiovascular diseases, such as cardiomyopathy [2], coronary artery diseases [3] and peripheral vascular diseases [4]. However, essential hypertension is of unknown origin and is characterized as a multifactorial disease involving genetic and environmental factors [5]. Familial and twin studies show that 30%-50% of the phenotypic variation of blood pressure (BP) is attributable to genetic heritability [6]. This implies that the contributions of genetic determinants to the development of hypertension are significant and elements on our genome may predispose some people to develop hypertension [7].

**Fig 1:**
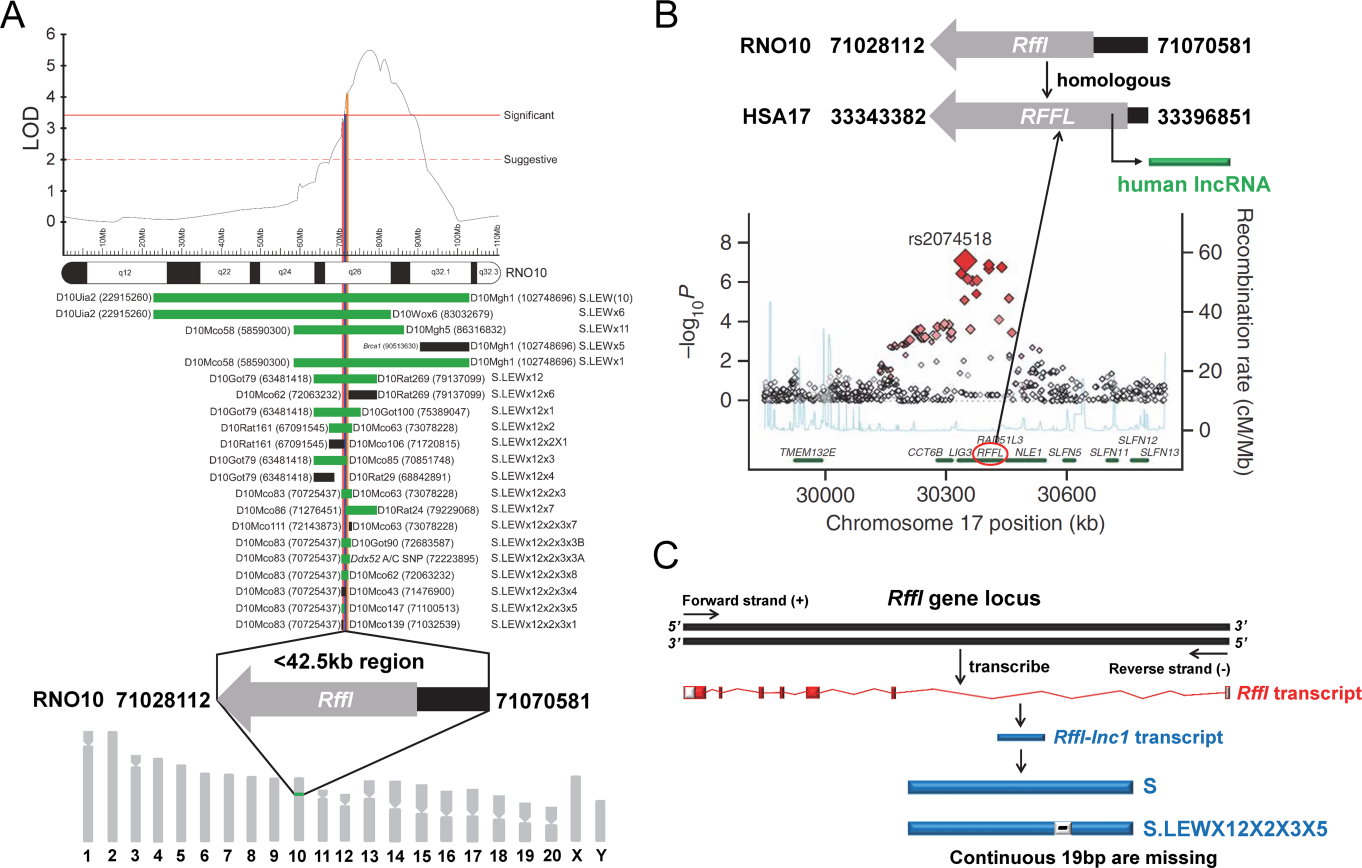
19bp sequence variation within *Rffl-lnc1* located in a <42.5kb QTL region. (A) A <42.5kb region with a single protein-coding gene, *Rffl*, was previously reported as a blood pressure quantitative trait locus (BP QTL) through high resolution mapping studies. The LOD plot is adapted from Saad Y et al [21]. (B) Short QT-interval syndrome was associated with multiple minor alleles in human *RFFL* gene with a human long non-coding RNA located in its 5’-UTR intronic region. The association plot is adapted from Newton-Cheh C et al [26]. (C) A novel rat long non-coding RNA, *Rffl-lnc1*, was identified within *Rffl* 5’-UTR intronic locus and there was a continuous 19bp sequence variation within *Rffl-lnc1* between the Dahl S rat and the S.LEW congenic strain (S.LEWX12X2X3X5). Rat RGSC3.4 assembly and human hg19 assembly were used for annotation.

Over the past 50 years, several animal models of essential hypertension, predominantly in the rat, have been developed as valuable tools to study the genetic factors associated with hypertension [8]. One such tool generated from our laboratory is the inbred Dahl salt-sensitive (S) rat. The S rat develops hypertension even on a low-salt diet but develops more severe hypertension when fed with a high-salt diet. Using this rat strain, we and others have applied classic genetic approaches of linkage followed by substitution mapping to locate regions of its genome as quantitative trait loci (QTLs) that are inherited causes of hypertension [9–19]. As relevant to the current study, we have previously mapped one such BP QTL on rat chromosome 10 by linkage followed by the construction and characterization of a custom series of congenic strains which contained introgressing genomic segments of normotensive Lewis rat (LEW) on the genetic background of the S rat. The mapped locus was within a <42.5kb region and reported as a quantitative trait locus (QTL) for BP as well as cardiac QT-interval [20–25] (Fig 1A). LEW alleles within the <42.5kb region significantly shortened QT-interval and increased blood pressure of the hypertensive S rat [20]. Interestingly, a large meta-analysis of three genome-wide association studies (GWAS) using 13,685 individuals reported that the region homologous to the rat 42.5kb region in humans, which lies on human chromosome 17, has multiple minor alleles that are reportedly associated with shorter QT-intervals [26] (Fig 1B). Of notable interest, nearly 30% of individuals in the GWAS study also had hypertension [26]. A second GWAS further confirmed the association of this locus to QT-interval [27]. Collectively, these observations suggest that the critical region in focus for the current report is of significance in the cardiovascular health of two mammalian species, the rat and human.

The <42.5kb critical region in rats contains a single protein coding gene, *Rffl*, which is without any exonic variants. The region also contains a novel long non-coding RNA (lncRNA), named *Rffl-lnc1*, located within *Rffl* 5’-UTR intronic region. *Rffl-lnc1* harbors a 19bp indel polymorphism between the S (+19bp) and the S.LEW congenic strain (-19bp), which were the two strains used to map this locus (Fig 1C). Based on this observation, we hypothesized that *Rffl-lnc1* is a genetic determinant of QT-interval and blood pressure.

To test this hypothesis, using the CRISPR/Cas9 technology, a panel of *Rffl-lnc1* disruption models was developed on the genomic background of the Dahl S rat. These models harbored varied disruptions around the critical 19bp region. The disruption of *Rffl-lnc1* significantly shortened QT-interval and increased blood pressure of the S rat, suggesting an important role of *Rffl-lnc1* in regulating cardiovascular function. To further evaluate the specific effect of the 19bp indel polymorphism within the *Rffl-lnc1* locus, a 19bp knock-in rescue model was developed on the genomic background of the S.LEW congenic strain using the CRISPR/Cas9 technology. The 19bp insertion successfully corrected the aberrant short QT-interval phenotype and lowered blood pressure of the S.LEW congenic strain, demonstrating that the 19bp indel polymorphism within *Rffl-lnc1* is an inherited genetic variation responsible for regulating cardiovascular disease in the rat. Further, our study has demonstrated that among all the variants located within the <42.5kb QTL region, the 19bp polymorphism was sufficient to regulate both QT-intervals and blood pressure. Overall, this study is the first to precisely define the quantitative trait nucleotides within a long non-coding RNA as a genetic determinant of cardiovascular function and is also the first to apply both gene-disruption and knock-in strategies using the CRISPR/Cas9 based genome editing approaches for delineating a complex cardiovascular trait locus in a mammalian model.

## Results

### CRISPR/Cas9 based genetic disruption of *Rffl-lnc1* in Dahl S rat

**Fig 2:**
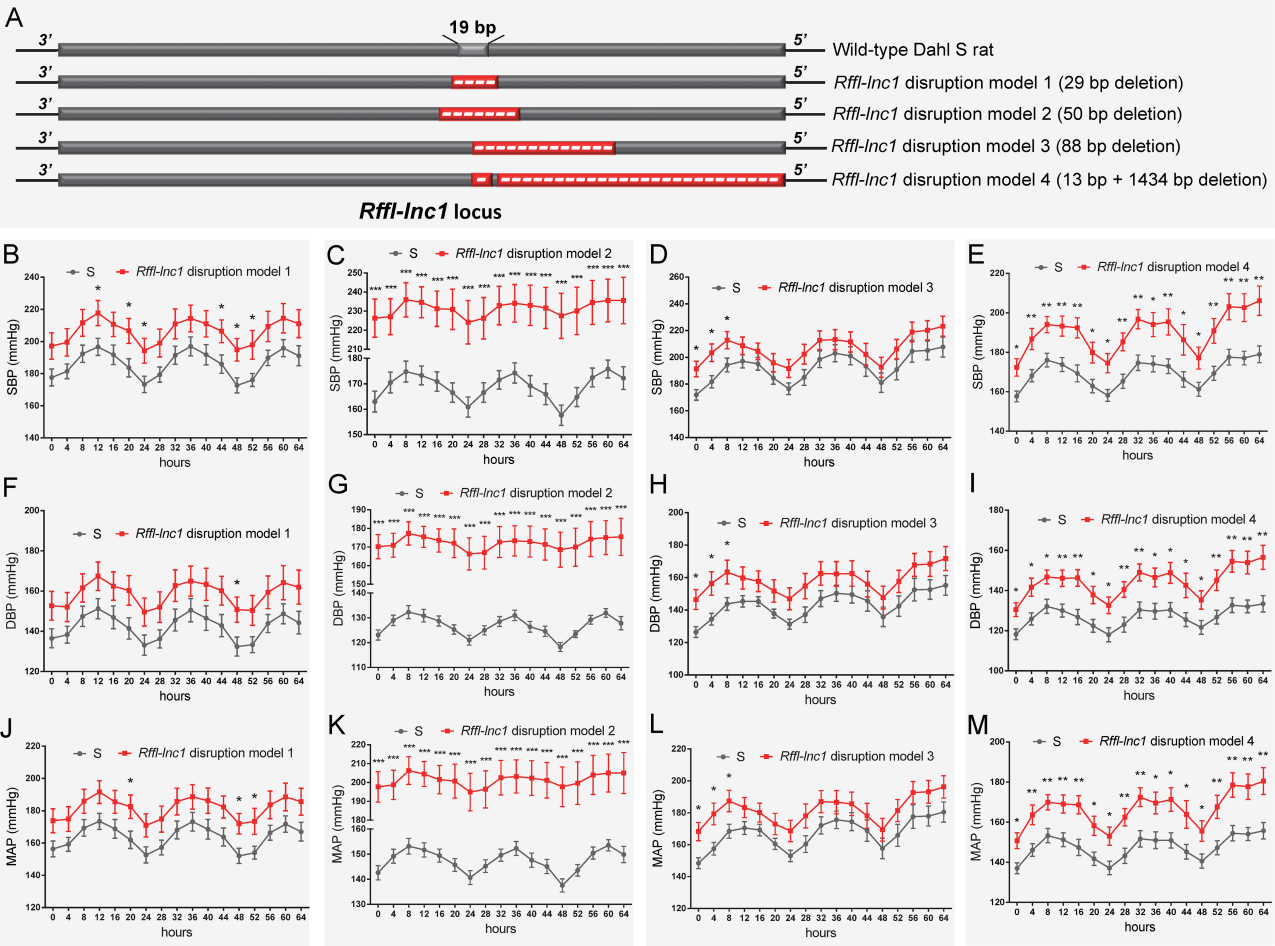
Elevated blood pressure in *Rffl-lnc1* disruption models compared with S rats. (A) Schematic of four *Rffl-lnc1* disruption models with different deletions around the 19bp locus. (B-E) Elevated systolic blood pressure (SBP) in *Rffl-lnc1* disruption models compared with the S rats. (F-I) Elevated diastolic blood pressure (DBP) in *Rffl-lnc1* disruption models compared with the S rats. (J-M) Elevated mean arterial pressure (MAP) in *Rffl-lnc1* disruption models compared with the S rats. At 6–7 weeks of age, rats were switched to a high-salt diet (2% NaCl) for 24 days, after which rats were surgically implanted with HD-S10 (previously C40) transmitters. Surgical rats were individually housed and allowed to recover for at least 3 days before recoding blood pressure. Data plotted was obtained by telemetry recording once every 5 minutes continuously and averaged for 4-hour intervals. Due to large difference of blood pressure between S rats and *Rffl-lnc1* disruption model 2, the discontinuous axis was used in panels C, G and K to correctly display circadian rhythm. All values are expressed as mean ± SEM. *: *P* < 0.05, **: *P* < 0.01, ***: *P* < 0.001. n = 7–9 rats/group.

To disrupt *Rffl-lnc1* on the genomic background of the Dahl S rat, a custom gRNA, r*Rffl*.g4, was designed to target the 19bp containing genomic segment. In vitro validation of r*Rffl*.g4 using mismatch detection assay confirmed its target efficiency (S1 Fig). Microinjection of gRNA and Cas9 mRNA into single cell embryos of the S rat followed by implantation into 6 pseudo-pregnant females resulted in a total of 67 pups. Genotyping and sequencing data showed that 21 out of these 67 pups were mutants within the *Rffl-lnc1* locus with disruptions both within and outside of the 19bp critical region. We used 4 founders with different deletions occurring within the 19bp locus for subsequent phenotypic studies (Fig 2A).

### Deteriorated cardiac function in *Rffl-lnc1* disruption models

**Fig 3:**
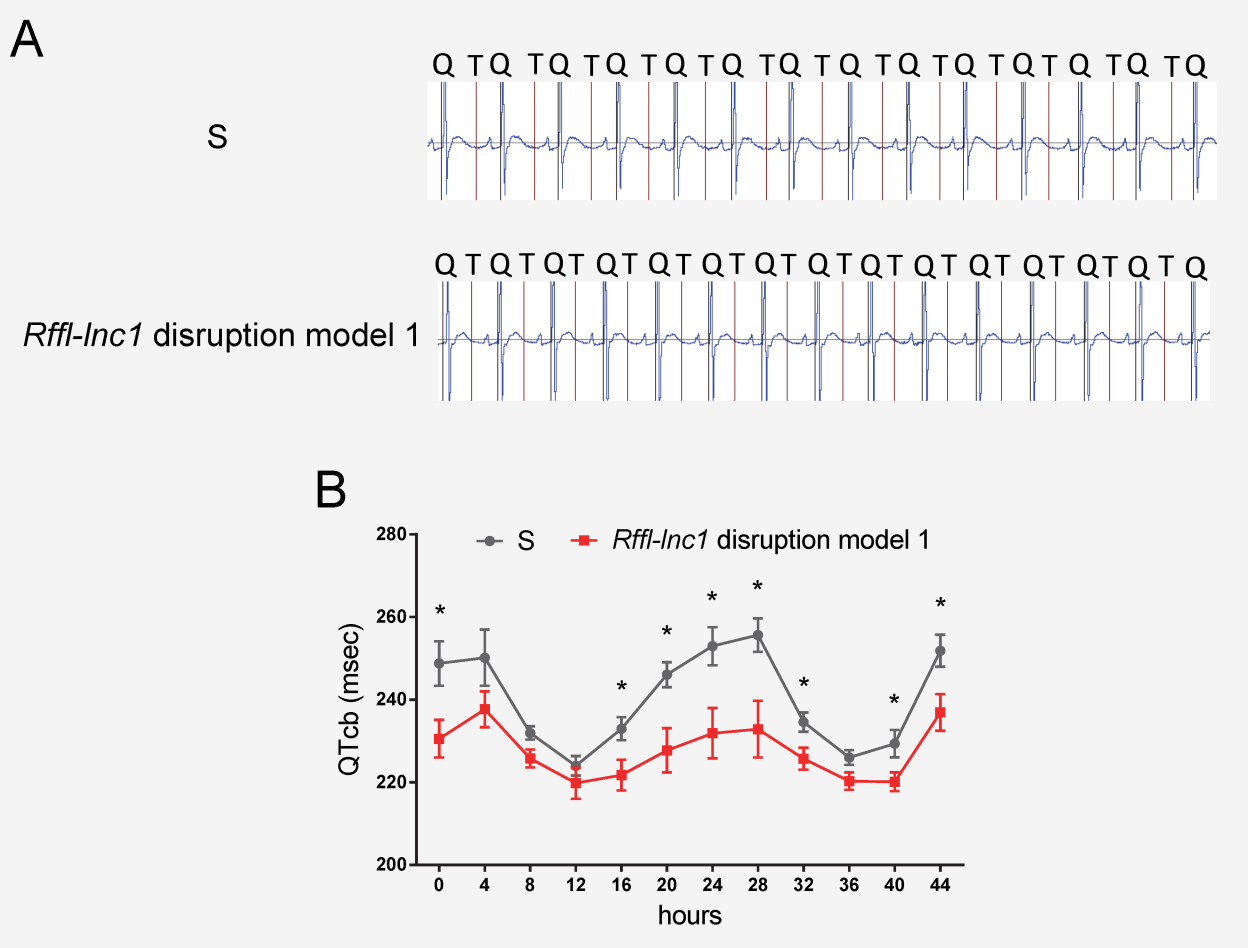
Shorter QT-intervals in *Rffl-lnc1* disruption model 1 compared with S rats. (A) Representative ECG recordings from individual S and *Rffl-lnc1* disruption model 1. (B) Shorter QT-intervals in *Rffl-lnc1* disruption model 1 compared with S rats (n = 5–6 rats/group). Experimental rats were maintained on low-salt diet after weaning and surgically implanted with CTA-F40 transmitters at about 18 weeks of age. Surgical rats were individually housed and allowed to recover for at least 3 days before ECG recording. Data plotted was obtained by telemetry recording once every 5 minutes continuously and averaged for 4-hour intervals. All values are expressed as mean ± SEM. *: *P* < 0.05.

All 4 *Rffl-lnc1* disruption models demonstrated elevated systolic, diastolic and mean arterial pressures compared to wild-type hypertensive S rats (Fig 2B–2M). Interestingly, these disruption models exhibited different levels of BP increasing effects (Fig 2B–2M). The heart/body weight ratios were also higher in *Rffl-lnc1* disruption models (S2 Fig), suggesting BP associated cardiac hypertrophy and potential dysfunction. To further assess cardiac function, we focused on QT-interval because shorter QT-intervals were reported to be associated with alleles within the homologous segment in humans as well as observed in our previous high resolution positional mapping study in rats [20, 26]. As shown in Fig 3A and 3B, the QT-intervals of the gene-edited *Rffl-lnc1* model were significantly shorter than that of the S rat. Collectively, these results demonstrate that *Rffl-lnc1* is a potential genetic determinant of blood pressure and QT-interval.

### Rapid amplification of cDNA ends (RACE) and secondary structure prediction for *Rffl-lnc1*

**Fig 4:**
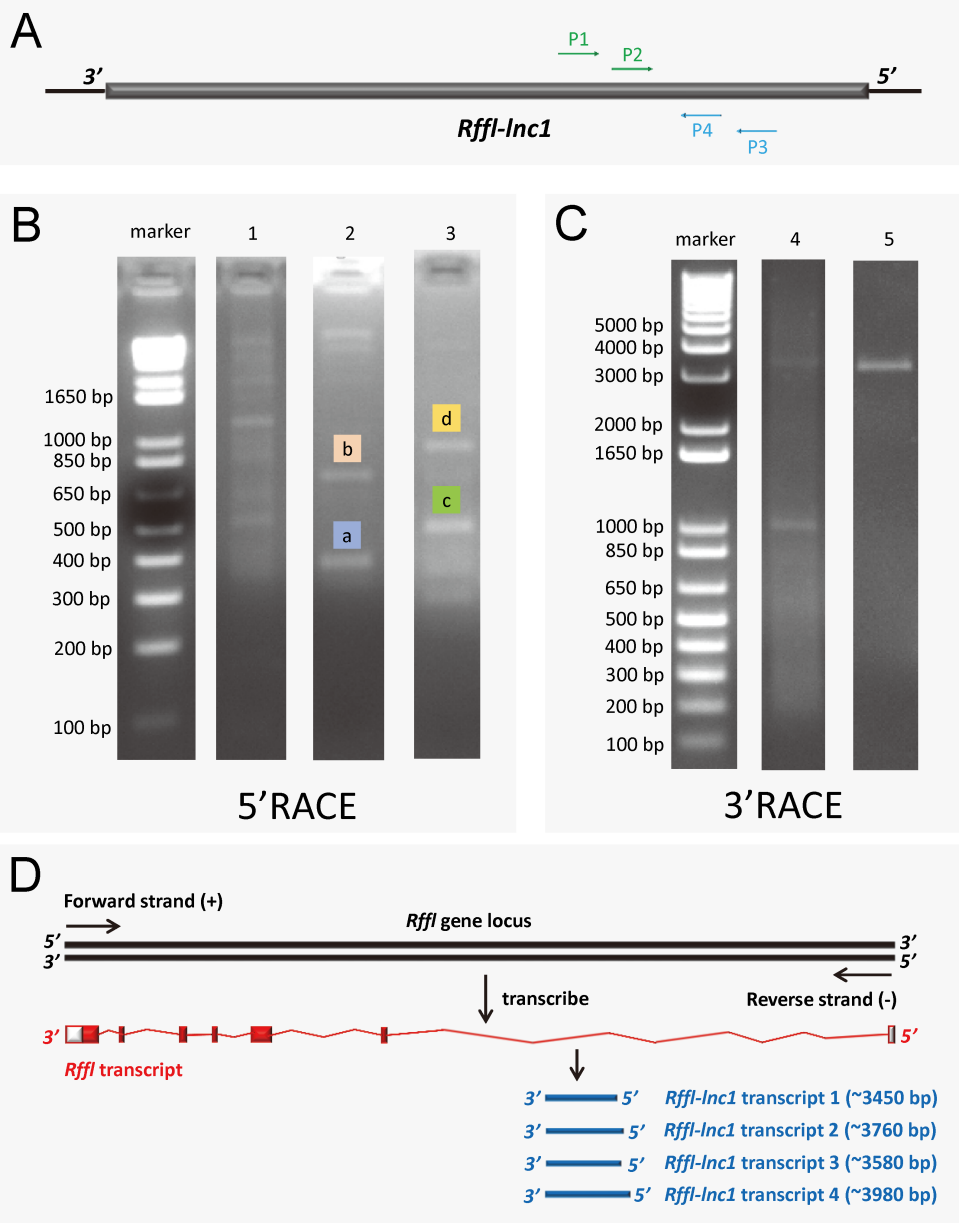
5' and 3' rapid amplification of cDNA ends (RACE) of *Rffl-lnc1*. (A) Primer design for 5’RACE and 3’RACE. P1 and P2 were designed for 5’RACE. P3 and P4 were designed for 3’RACE. (B) 5’RACE PCR amplification. Lane 1 shows the PCR products by using P1 and UPM to amplify 5’RACE cDNA. Lane 2 and 3 show the distinct 5’RACE PCR products a, b, c and d by using P2 and UPS to amplify the diluted PCR product from lane 1 (nested PCR). (C) 3’RACE PCR amplification. Lane 4 shows PCR products by using P3 and UPM to amplify 3’RACE cDNA. Lane 5 shows the distinct 3’RACE PCR product by using P4 and UPS to amplify the diluted PCR product from lane 4 (nested PCR). (D) The schematic showing four *Rffl-lnc1* transcripts. The full length sequences of *Rffl-lnc1* were obtained by in-fusion cloning of RACE products for sequencing.

**Fig 5:**
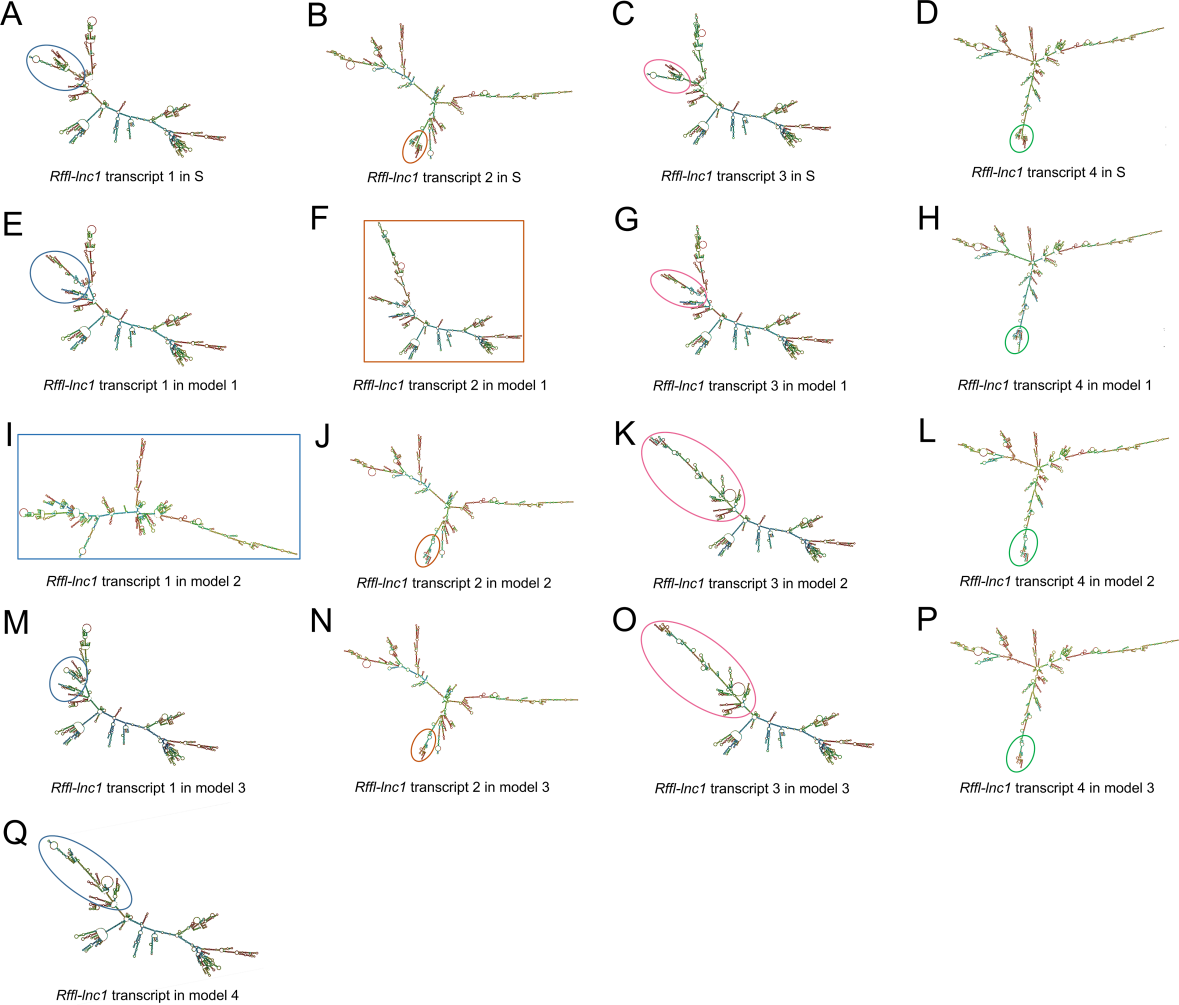
Bioinformatics prediction of *Rffl-lnc1* secondary structures in the S rat and *Rffl-lnc1* disruption models. (A-D) *Rffl-lnc1* secondary structures in wild-type S rats. (E-H) *Rffl-lnc1* secondary structures in *Rffl-lnc1* disruption model 1. (I-L) *Rffl-lnc1* secondary structures in *Rffl-lnc1* disruption model 2. (M-P) *Rffl-lnc1* secondary structures in *Rffl-lnc1* disruption model 3. (Q) *Rffl-lnc1* secondary structure in *Rffl-lnc1* disruption model 4. MFE (minimum free energy) secondary structures of *Rffl-lnc1* were predicted using RNAfold Webserver [28] (http://rna.tbi.univie.ac.at/cgi-bin/RNAWebSuite/RNAfold.cgi) with default parameters. Circles and rectangles point out the sites of structural differences, such as double helices, internal loops and stem loops, for each transcript. Large and clear secondary structures of each transcript in the S rat and disruption models are provided in S3–S19 Figs.

Since the annotation for the novel *Rffl-lnc1* was limited to a few base-pairs, we performed RACE experiments to ascertain its full sequence. 5’RACE amplifications using the primer P1 (Fig 4A) and Universal Primer A Mix (UPM) for the initial PCRs followed by nested PCR amplification using the primer P2 (Fig 4A) and Universal Primer Short (UPS) resulted in four 5’RACE products, labeled as a, b, c and d, in Fig 4B. Unlike 5’RACE, Fig 4C shows the unique 3’RACE product in lane 5, which was obtained using the primer P3 (Fig 4A) and UPM for the initial PCR followed by nested PCR amplification using P4 (Fig 4A) and UPS. Further characterization of these PCR products by sequencing confirmed the existence of four different isoforms of *Rffl-lnc1*, each with a different 5’ end. Each isoform contained a single-exon of more than 3000bp (Fig 4D). The secondary structures of these isoforms of *Rffl-lnc1* were predicted using RNAfold Webserver [28] (Fig 5). The 4 isoforms of *Rffl-lnc1* showed different secondary structures in the wild-type S rat (Fig 5A–5D). Interestingly, it was observed that sequence deletions around the critical 19bp region of *Rffl-lnc1* caused a range of perturbations of the secondary structures, such as double helices, internal loops and stem loops, of all the four isoforms in each of the gene-disruption models (Fig 5E–5Q). The most deleterious perturbation was that in *Rffl-lnc1* disruption model 4, wherein, due to the large deletion of the 5’ end of *Rffl-lnc1*, the secondary structural integrity was lost in all the isoforms except one (Fig 5Q). Secondary structures of *Rffl-lnc1* in all the disruption models appeared to correlate well with physiological impact. For instance, the secondary structure of *Rffl-lnc1* transcript 1 was drastically altered in *Rffl-lnc1* disruption model 2 compared to other models and the S rat (Fig 5A, 5E, 5I, 5M and 5Q), which correlated with a dramatic BP increasing effect observed in *Rffl-lnc1* disruption model 2 compared to other models (Fig 2). The correlation indicates a potential link between lncRNA structure and its physiological impact.

### CRISPR/Cas9 based targeted rescue of *Rffl-lnc1* in the S.LEW congenic strain

**Fig 6:**
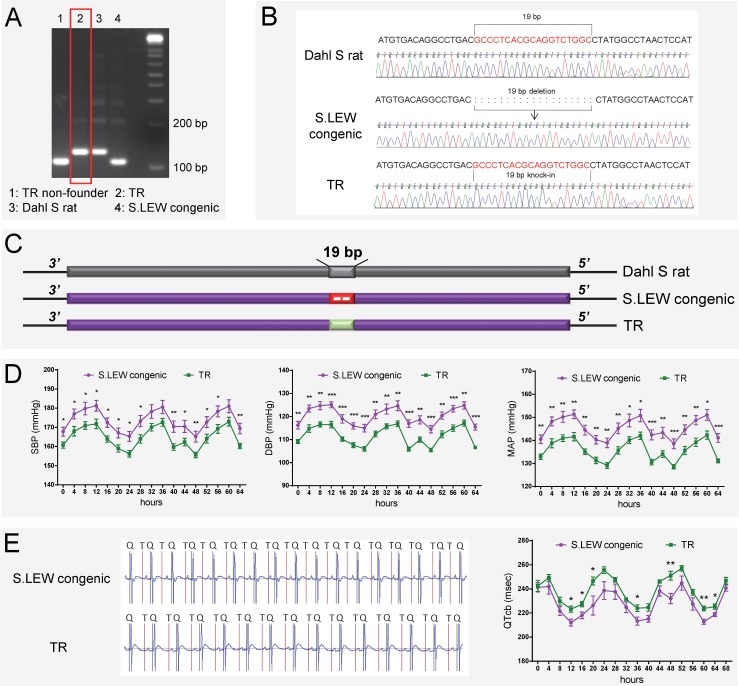
19bp knock-in targeted rescue (TR) of blood pressure and QT-interval in the S.LEW congenic rat. (A-C) Genotyping and sequencing showing successful 19bp knock-in in *Rffl-lnc1* of the S.LEW congenic rat. (D) Lower systolic, diastolic and mean arterial pressures in TR rats compared with wild-type S.LEW congenic rats (n = 8 rats/group). (E) Improved QT-intervals in TR rats compared with wild-type S.LEW congenic rats (n = 5 rats/group). Experimental rats were maintained on low-salt diet after weaning and surgically implanted with HD-S10 (previously C40) transmitters for BP recording at about 13 weeks of age. At about 16 weeks of age, the experimental rats were surgically implanted with CTA-F40 transmitters for ECG recording. Surgical rats were individually housed and allowed to recover for at least 3 days before BP and ECG recording. BP and ECG data plotted were obtained by telemetry recording once every 5 minutes continuously and averaged for 4-hour intervals. All values are expressed as mean ± SEM. *: *P* < 0.05, **: *P* < 0.01, ***: *P* < 0.001.

The above evidence obtained with gene-disruption models, albeit strong, does not directly test causality for the 19bp as the naturally occurring quantitative trait nucleotides within *Rffl-lnc1* affecting cardiovascular function. To directly evaluate the contribution of the 19bp indel polymorphism on cardiovascular function, we further used the CRISPR/Cas9 system to generate a targeted knock-in rescue model by precisely inserting the 19bp into the *Rffl-lnc1* locus of the S.LEW congenic strain. A total of 73 pups were born after the microinjection of r*Rffl*.g4, Cas9 mRNA and donor oligonucleotide into single cell embryos of the S.LEW congenic strain, followed by implantation into 7 pseudo-pregnant females. Genotyping and sequencing validations identified 3 successful 19bp knock-in founders (Fig 6A–6C).

**Fig 7:**
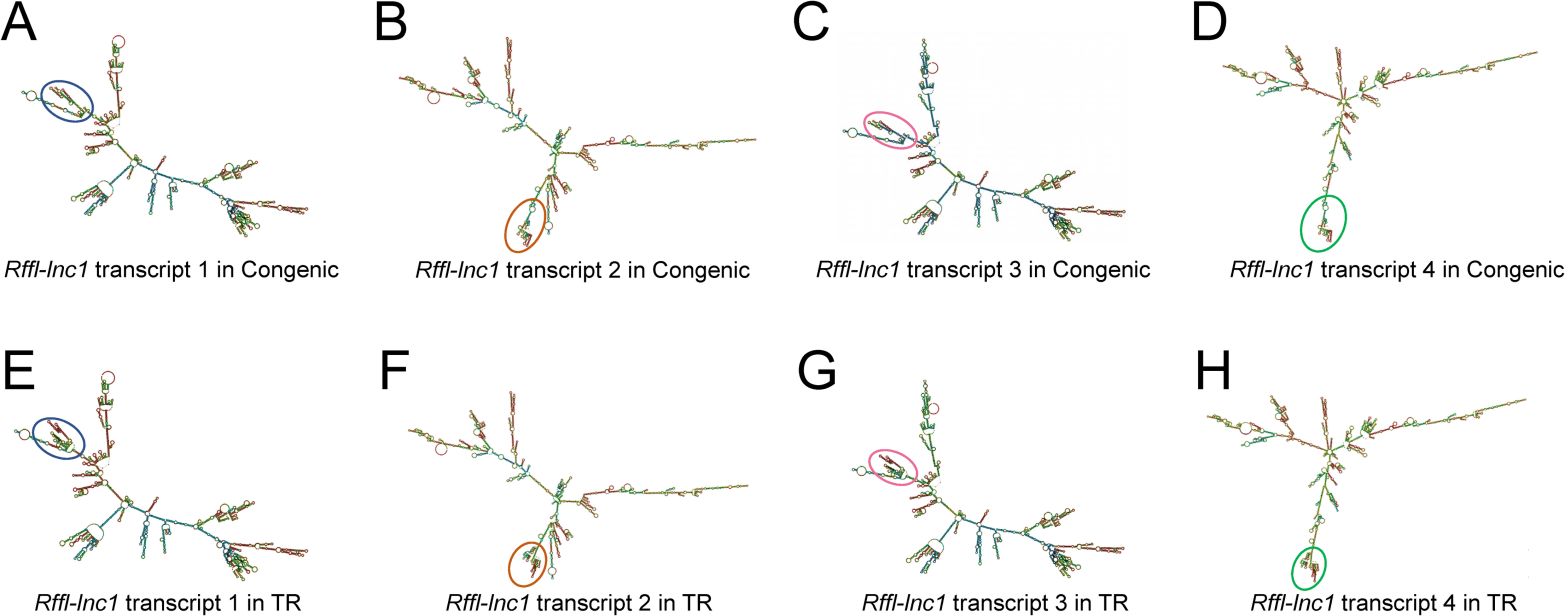
Bioinformatics prediction of *Rffl-lnc1* secondary structures in the S.LEW congenic rat and 19bp knock-in targeted rescue (TR) model. (A-D) *Rffl-lnc1* secondary structures in the S.LEW congenic rat. (E-H) *Rffl-lnc1* secondary structures in the TR model. MFE (minimum free energy) secondary structures of *Rffl-lnc1* were predicted using RNAfold Webserver [28] (http://rna.tbi.univie.ac.at/cgi-bin/RNAWebSuite/RNAfold.cgi) with default parameters. Circles and rectangles point out the sites of structural differences, such as double helices, internal loops and stem loops, for each transcript. Large and clear secondary structures of each transcript in the S.LEW congenic rat and the TR model are provided in S20–S27 Figs.

Knock-in rescue rats exhibited significantly lower systolic, diastolic and mean arterial pressures compared to wild-type S.LEW congenic rats (Fig 6D). Echocardiac evaluation demonstrated that knock-in rescue rats tended to have lower relative wall thickness and exhibited significantly improved cardiac function and contractility as evidenced by significantly lower MPI and increased FS/MPI, respectively (S1 Table). Moreover, short QT-intervals were also improved in targeted knock-in rescue rats compared to wild-type congenic rats (Fig 6E). Fig 7 catalogs the structural differences of the four *Rffl-lnc1* transcripts between targeted knock-in rescue rats and wild-type S.LEW congenic rats, which reflect the direct rescue status of the transcripts. These results demonstrate that the 19bp indel polymorphism is specifically responsible for functioning as quantitative trait nucleotides within four isoforms of a long non-coding RNA that are involved in cardiovascular regulation.

### The 19bp polymorphism is necessary and sufficient for cardiovascular regulation

**Fig 8:**
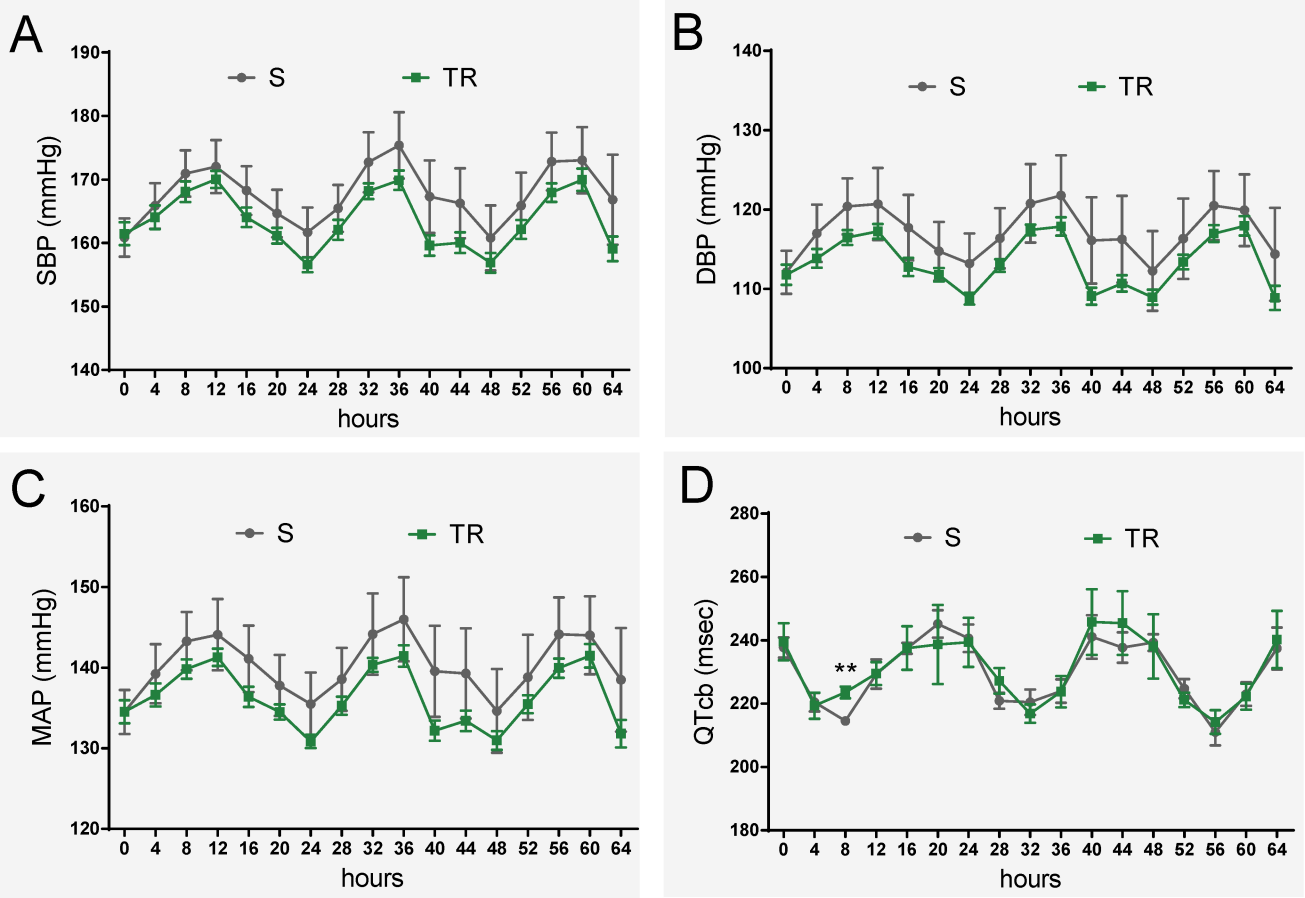
Evaluation of 19bp sufficiency in cardiovascular regulation. (A-C) No significant difference in blood pressure between S and TR rats (n = 8 rats/group) (D) No significant difference in QT-interval between S and TR rats (n = 5 rats/group). Experimental rats were maintained on low-salt diet after weaning and surgically implanted with HD-S10 (previously C40) transmitters for BP recording at about 11 weeks of age. At about 13 weeks of age, the experimental rats were surgically implanted with CTA-F40 transmitters for ECG recording. Surgical rats were individually housed and allowed to recover for at least 3 days before BP and ECG recording. BP and ECG data plotted were obtained by telemetry recording once every 5 minutes continuously and averaged for 4-hour intervals. All values are expressed as mean ± SEM. **: *P* < 0.01.

The critical <42.5kb QTL region contains 171 variants including the continuous 19bp variation [20]. To evaluate whether the 19bp are sufficient to regulate cardiac function, we further compared the phenotypes between S and targeted knock-in rescue rats. Interestingly, knock-in rescue rats demonstrated no differences in blood pressure and QT-interval compared to S rats (Fig 8). No significant differences in echocardiographic parameters were seen (S2 Table). These results demonstrate that the other variants within the QTL region are not contributing to the QTL effect and importantly, that the 19bp indel polymorphism within the previously resolved <42.5kb QTL region is necessary and sufficient to demonstrate the full effect of the QTL region independent to the other allelic variations within the QTL segment.

## Discussion

The search for inherited factors responsible for blood pressure and cardiovascular traits has been dominated by genome wide-association studies in humans [29–37] and positional mapping approaches in model organisms [38, 39]. The former has resulted in the identification of hundreds of genomic variants and the latter has been successful in mapping genomic segments of mammalian model organisms, predominantly the rat. Data from both of these approaches is consistently pointing to variants in non-coding elements as being the most prevalent signals for associations to blood pressure. Beyond such associations, there is a wide gap in our understanding of the precise identities of the allelic variants of non-coding elements and their functional link to cardiovascular health and disease.

The present work, which is backed by persistent and systematic mapping [20–25], for the first time, identifies a 19bp indel polymorphism as the precise variation within a novel lncRNA, *Rffl-lnc1*, which is necessary for BP regulation. This is also the first study wherein the state-of-the-art genome engineering using the CRISPR/Cas9 based genome targeted rescue strategy is applied beyond a mapping approach to identify quantitative trait nucleotides responsible for cardiovascular effects.

The impetus for focusing on non-coding elements within our previously mapped <42.5kb genomic region, was the observation that the region contained only a single protein-coding gene, *Rffl* (Fig 1A), which did not contain any exonic variants. More interestingly, this short <42.5kb genomic segment was also involved in the regulation of QT-intervals [20], which further translationally supported a large meta-analysis of human GWAS on its homologous genomic region on human chromosome 17 [26] (Fig 1B).

There were a total of 171 variants to consider within the QTL region [20], among which a long stretch of contiguous 19bp indel polymorphism caught our attention. At this point, we were also developing a first-generation catalog of rat lncRNAs involved in BP regulation [40], through which we identified a novel lncRNA, named *Rffl-lnc1*. As a happenstance, the contiguous 19bp indel polymorphism overlapped the available annotation for *Rffl-lnc1* (Fig 1C). Therefore, we chose to prioritize the 19bp indel polymorphism and focused on testing the hypothesis that this particular variation is responsible for the QTL effect. In obtaining positive results that prove our hypothesis, our present study has: (1) Further improved the high resolution mapping from the <42.5kb QTL to 19bp; (2) Eliminated the remainder of the QTL region as not independently contributing to the QTL effect; (3) Defined the QTL as being due to the quantitative nucleotide variation within a novel, functional long non-coding *Rffl-lnc1*; (4) Determined the function of *Rffl-lnc1* as being important for two cardiovascular complex traits, i.e., blood pressure and cardiac QT-interval; and (5) Identified the involvement of at least 4 isoforms of *Rffl-lnc1* with disparate secondary structures to be considered as being important for further functional cardiovascular regulatory studies.

Considering that data from the targeted rescue model is sufficient evidence to assign causality for the 19bp indel polymorphism, it may appear that the gene-disruption models were not that important. On the contrary, the disruption models were quite informative. The targeted disruption models of *Rffl-lnc1* served as important evidence for suspecting the role of *Rffl-lnc1* in regulating cardiovascular functions. We also predicted the secondary structures in the disruption models and surprisingly, large structural changes were observed in *Rffl-lnc1* transcripts of different models due to different sequence deletions occurring around the 19bp locus (Fig 5). For example, *Rffl-lnc1* transcript 1 in *Rffl-lnc1* disruption model 2 is drastically different compared to that in other three disruption models and the S rat (Fig 5A, 5E, 5I, 5M and 5Q), which corresponded to much higher BP increasing effects in the model 2 compared to the other 3 models (Fig 2). Due to the large deletion occurring in the 5’ end of *Rffl-lnc1* in *Rffl-lnc1* disruption model 4, only one isoform appears to remain intact in this model (Fig 5Q). Interestingly, the insertion of only 19bp in the targeted rescue model caused discernible structural modifications, such as double helices, internal loops and stem loops, in all the transcripts (Fig 7). The correlation between molecular lncRNA isoform structures and their physiological effects further provides evidence to the point that different lncRNA isoforms may have varied physiological impact. Whether all or only some of these 4 *Rffl-lnc1* transcripts are required for the observed physiological effects remains to be determined. It is also unknown whether additional, yet undetected, *Rffl-lnc1* transcripts exist. Nevertheless, the role of the identified 19bp indel polymorphism is clearly established as being functional at least through one (or more) of the isoforms of *Rffl-lnc1*. Also, these first-generation genome-engineered targeted disruption models for a lncRNA serve as excellent tools for further studies.

The most rigorous test for assigning the ‘quantitative trait nucleotide’ status to an allelic variant on the genome is to demonstrate a direct cause-effect relationship between the allelic variation and an alteration in a physiological trait using a targeted rescue approach in a model organism [38, 41]. To apply this level of rigor was impossible in rat models until the CRISPR/Cas9 technology became applicable to the rat model. Therefore, we have taken advantage of this technological advancement to further test whether the 19bp served as quantitative trait nucleotides. Our results demonstrate that by restoring *Rffl-lnc1* with the 19bp insertion, the rescue model lowered hypertension and corrected the short QT-interval phenotype. Thus, the 19bp indel polymorphism is hitherto defined as quantitative trait nucleotides for cardiovascular regulation of blood pressure and cardiac QT-intervals.

The next ambiguity pertained to the contribution of the remainder of the QTL region, because the experimental design of comparing the targeted rescue model, which was developed on the genomic background of the S.LEW congenic strain, with the congenic strain as the control strain, was not informative for the contributions, if any, of the remainder of the variants within the QTL region. To overcome this ambiguity, we used the experimental design of comparing the targeted rescue model with the S rat, whereby, the precise contribution of the remainder of the QTL region (without the 19bp variation) could be assessed. The result from this study wherein there were no phenotypic differences between the targeted rescue model and the S rat (Fig 8), suggests two possibilities. The first possibility is that other variants are not important and the 19bp play an exclusive role in cardiovascular regulation. The second possibility is that other variants may be in epistasis, whereby they may not be able to exert their effects independent of the 19bp. Either way, this provides evidence to indicate that the 19bp polymorphism is critical for the QTL effect and that the remainder of the QTL region is relatively insignificant in independently contributing to the QTL effect.

Although our study identifies QTNs for arterial blood pressure, it does not specifically address whether the QT interval shortening in the Dahl S and the rescue with the transgenic 19bp insertion is a direct consequence of changes in the cardiac conduction system, or due to a primary modification of neural autonomic pathways that alter electrogenic cardiac functions, or due to the result of cardiac hypertrophy as a secondary consequence to the chronic elevation of BP (which is well known to alter electrical conduction intervals [42, 43]). Teasing apart these coupled features requires combinatorial experimental designs of using the genetic models developed through our study and pharmacological approaches.

Our previous study provided evidence for this <42.5kb QTL region as also being important for the regulation of tumorigenesis [44], therefore our future study will further investigate the roles of *Rffl-lnc1* and the 19bp within it in the process of tumorigenesis using our disruption and rescue models. As we mentioned earlier, a homologous locus on human chromosome 17 of this <42.5kb critical region on rat chromosome 10 was studied in a large meta-analysis of human GWAS, showing multiple alleles near human *RFFL* gene were associated with the short QT-interval syndrome [26] (Fig 1B) as confirmed by our congenic and transgenic rat models. Although hypertension was not pointed out to be associated with this locus in humans, it is reported that nearly 30% of individuals in this GWAS study had hypertension [26]. Therefore, our study serves as the basis to consider hypertension as an important co-segregating phenotype along with QT-interval in humans. Further the delineation of a lncRNA as the quantitative trait gene within the rat locus prompts the consideration of a similar lncRNA within the homologous human region with reported association for QT-interval. To this point, it is interesting to note that a human lncRNA does exist within the 5’-UTR intronic region of the human *RFFL* gene (Fig 1B). Given the rat data, our study may serve as a translational foundation for considering this human lncRNA as a candidate regulator for cardiovascular diseases.

### Conclusions

This study has contributed to the advancement of QTL mapping in the rat for cardiovascular phenotypes in general by pinpointing the quantitative trait nucleotides underlying a QT-interval and a BP QTL. It is also the first report of a polymorphism detected within a long non-coding RNA as a candidate gene verified within a mammalian QTL. The translational significance of the study is that it provides additional confirmatory evidence for the homologous region in humans detected to be associated with QT-interval. Due to the lack of sequence conservation between rats and humans, the precise polymorphisms within the homologous *Rffl-lnc1* locus may or may not exist in humans. The relevance of this positional cloning study is therefore that the results obtained by mapping a locus in the rat provide a functional basis to assess similar effects of variants within the lncRNA as candidates within the homologous region in humans reported by GWAS for QT-interval.

## Materials and methods

### Animals and diet

All animal procedures and protocols described in this study were approved by the University of Toledo Institutional Animal Care and Use Committee. Animal experiments were performed in accordance with the Guide for the Care and Use of Laboratory Animals. The inbred Dahl salt-sensitive (SS/Jr or S) rat strain was from stocks maintained in our animal facility at our institution. Rats were weaned at 28–30 days of age and fed with a low-salt diet (0.3% NaCl, TD 7034, Harlan Teklad). High-salt diet (2% NaCl, TD 94217, Harlan Teklad) was used for experiments involving a high-salt regimen. Only male rats were used for the current study, in order to match the blood pressure QTL inference drawn from the previous study [20] conducted using male rats. In each phenotypic study, any different experimental rat groups were concomitantly bred and co-housed to minimize environmental effects.

### Generation of a CRISPR/Cas9 targeted disruption and rescue model

Guide RNAs (gRNAs) were designed to target the 19bp locus within *Rffl-lnc1* (Genome Engineering and iPSC Center, Washington University, St. Louis, MO). Bioinformatics analysis was performed to detect potential off-target sites of all gRNA candidates on the rat genome. The gRNA, r*Rffl*.g4 (AAGCCATGGAGTTAGGCCATNGG), which had minimum off-target potential based on homology, was further validated in rat C6 cells. The gRNA, r*Rffl*.g4, was chosen for the transgenesis in generating both disruption and knock-in rescue models. Additionally, a donor oligonucleotide (CACCACCCCAGCAGCTCCTGTTGAGCACTGCAGCGGCCTCATCCATGTGACAGGCCTGACGCCCTCACGCAGGTCTGGCCTATGGCCTAACTCCATGGCTTTCCAAGTGCTGGAAGTTCCCCAGGCGACATTCAGTGTC), which contains the 19bp sequence, was designed for the knock-in rescue model.

Oocyte microinjection was conducted at the University of Michigan Transgenic Animal Model Core (Ann Arbor, MI). For the disruption model, a mixture of r*Rffl*.g4 (2.5 ng/μl) and Cas9 mRNA (5 ng/μl) was injected into one-cell stage Dahl salt-sensitive (S) rat embryos. Microinjected embryos were implanted into 6 pseudo-pregnant Sprague-Dawley female rats and a total of 67 pups were born. For the knock-in rescue model, a mixture of r*Rffl*.g4 (2.5 ng/μl), Cas9 mRNA (5 ng/μl) and the donor oligonucleotide (10 ng/μl) was injected into one-cell stage S.LEW congenic strain embryos. Microinjected embryos were implanted into 7 pseudo-pregnant Sprague-Dawley female rats and a total of 73 pups were born. At 14 days of age, tail tip biopsies were collected from transgenic pups for extracting genomic DNA. Three different primer sets were used for initial genotyping. The forward (F) and reverse (R) sequences of these three primer sets (1, 2, 3) are: 1-F: AGCAGCTCCTGTTGAGCACT; 1-R: GAACTTCCAGCACTTGGAAAGC; 2-F: ACTGCCCTGAACCAAACCTG; 2-R: ACTTGGAAAGCCATGGAGTTAG; 3-F: ATGCAGACGATTTCTGACAGC; 3-R: ATCCCTGAGGGCTTTTCTACA. Due to large deletions in *Rffl-lnc1* disruption model 4, the forward (ATGCAGACGATTTCTGACAGC) and reverse (GGTCTTCACTCTCCAGAATATG) primers were used for further genotyping. After breeding all the potential founders to homozygotes, the PCR products of the genotyping from the homozygotes were sent for sequencing validation (Eurofins MWG Operon, https://www.eurofinsgenomics.com/en/home.aspx) and sequencing data was analyzed using Sequencher 4.10.1. The homozygotes of disruption and knock-in models were used for subsequent phenotypic studies.

### Blood pressure measurements by radiotelemetry

Blood pressure was recorded and analyzed using radiotelemetry transmitters (HD-S10 or previously C40), receivers and software from Data Sciences International, as described previously [21]. The specific details on the age of the rat and type of diet used in each study are provided in the legend to each figure.

### Biotelemetry electrocardiogram (ECG)

ECG data was collected and analyzed using CTA-F40 transmitters, receivers and software from Data Sciences International. Briefly, the transmitters were surgically implanted into the peritoneal cavity of rats under anesthesia and transmitter electrodes were arranged in Lead II configuration. ECG data was collected at 5-minute intervals and analyzed using Ponemah v.5.2 (Data Sciences International). Bazett’s formula was used as the standard correction method for normalizing QT-intervals specifically for rats. The specific details on the age of the rat and type of diet used in each study are provided in the legend to each figure.

### Rapid amplification of cDNA ends (RACE)

Total RNA was extracted from heart tissues of the Dahl S rat using the TRIzol reagent (Life Technologies) according to the manufacturer’s instructions. The integrity and concentration of the RNA was assessed by gel electrophoresis and NanoDrop 2000 (Thermo Scientific). 5’RACE and 3’RACE procedures were performed according to the SMARTer RACE 5’/3’ Kit (Clontech) protocol. Briefly, about 5μg RNA was used for making 5’RACE and 3’RACE cDNA, respectively. For 5’RACE amplification, P1 (GATTACGCCAAGCTTACCCCAGCAGCTCCTGTTGAGCACT) and Universal Primer A Mix (UPM) were used for initial PCR amplification according to Program 1 (touchdown PCR) in the protocol. Using the diluted (50X) PCR product from the previous step, P2 (GATTACGCCAAGCTTTGGGCACAATAGCTTGGCTTTTATGGAC) and Universal Primer Short (UPS) were used for the nested PCR according to Program 2 in the protocol to obtain the 5’RACE products for the following characterization. For 3’RACE amplification, P3 (GATTACGCCAAGCTTAACCATTCAGGAAGCCACAGGCCTTCC) and UPM were used for initial PCR amplification according to Program 1 (touchdown PCR) in the protocol. Using the diluted (50X) PCR product from the previous step, P4 (GATTACGCCAAGCTTGTCCCGCCTTCCTATTTTCCAGATGAGG) and UPS were used for the nested PCR according to Program 2 in the protocol to obtain the 3’RACE product for the following characterization. The 5’RACE and 3’RACE products were further characterized following the steps of gel extraction and in-fusion cloning in the protocol. The cloned inserts were PCR amplified and sent for sequencing (Eurofins MWG Operon, https://www.eurofinsgenomics.com/en/home.aspx) and sequencing data was analyzed using Sequencher 4.10.1.

### Echocardiography

Left ventricular function and geometry of Dahl S rats, S.LEW congenic rats and 19bp targeted knock-in rescue model were evaluated by echocardiography, as described previously [45, 46]. The specific details on the age of the rat and type of diet used in each study are provided in S1 and S2 Tables.

### Statistical analysis

Two-tailed Student’s t-test was used for statistical analyses. Data are presented as mean ± SEM. A p-value of <0.05 was considered to be statistically significant.

## Supporting information

S1 Fig In vitro validation of r*Rffl*.g4 using mismatch detection assay confirming cutting activity. Rat C6 cells were transfected with gRNA and Cas9 encoding plasmids. Three days post-transfection, genomic DNA was harvested and used as template for the mismatch detection assay using the T7E1 enzyme. The uncleaved (508bp) amplicon and cleaved products (341bp and 167bp) are indicated. Cleavage products indicate active cleavage by r*Rffl*.g4 with Cas9.
(DOCX)

S2 Fig The heart weight/body weight ratios were higher in *Rffl-lnc1* disruption models compared with S rats. (A) *Rffl-lnc1* disruption model 1 versus S. (B) *Rffl-lnc1* disruption model 2 versus S. (C) *Rffl-lnc1* disruption model 3 versus S. (D) *Rffl-lnc1* disruption model 4 versus S. Hearts were collected within one week after blood pressure data (Fig 2) was collected. All values are expressed as mean ± SEM. n = 7–9 rats/group.
(DOCX)

S3 Fig Secondary structure of *Rffl-lnc1* transcript 1 in Dahl S rat. (DOCX)

S4 Fig Secondary structure of *Rffl-lnc1* transcript 2 in Dahl S rat. (DOCX)

S5 Fig Secondary structure of *Rffl-lnc1* transcript 3 in Dahl S rat. (DOCX)

S6 Fig Secondary structure of *Rffl-lnc1* transcript 4 in Dahl S rat. (DOCX)

S7 Fig Secondary structure of *Rffl-lnc1* transcript 1 in *Rffl-lnc1* disruption model 1. (DOCX)

S8 Fig Secondary structure of *Rffl-lnc1* transcript 2 in *Rffl-lnc1* disruption model 1. (DOCX)

S9 Fig Secondary structure of *Rffl-lnc1* transcript 3 in *Rffl-lnc1* disruption model 1. (DOCX)

S10 Fig Secondary structure of *Rffl-lnc1* transcript 4 in *Rffl-lnc1* disruption model 1. (DOCX)

S11 Fig Secondary structure of *Rffl-lnc1* transcript 1 in *Rffl-lnc1* disruption model 2. (DOCX)

S12 Fig Secondary structure of *Rffl-lnc1* transcript 2 in *Rffl-lnc1* disruption model 2. (DOCX)

S13 Fig Secondary structure of *Rffl-lnc1* transcript 3 in *Rffl-lnc1* disruption model 2. (DOCX)

S14 Fig Secondary structure of *Rffl-lnc1* transcript 4 in *Rffl-lnc1* disruption model 2. (DOCX)

S15 Fig Secondary structure of *Rffl-lnc1* transcript 1 in *Rffl-lnc1* disruption model 3. (DOCX)

S16 Fig Secondary structure of *Rffl-lnc1* transcript 2 in *Rffl-lnc1* disruption model 3. (DOCX)

S17 Fig Secondary structure of *Rffl-lnc1* transcript 3 in *Rffl-lnc1* disruption model 3. (DOCX)

S18 Fig Secondary structure of *Rffl-lnc1* transcript 4 in *Rffl-lnc1* disruption model 3. (DOCX)

S19 Fig Secondary structure of *Rffl-lnc1* transcript in *Rffl-lnc1* disruption model 4. (DOCX)

S20 Fig Secondary structure of *Rffl-lnc1* transcript 1 in S.LEW congenic strain. (DOCX)

S21 Fig Secondary structure of *Rffl-lnc1* transcript 2 in S.LEW congenic strain. (DOCX)

S22 Fig Secondary structure of *Rffl-lnc1* transcript 3 in S.LEW congenic strain. (DOCX)

S23 Fig Secondary structure of *Rffl-lnc1* transcript 4 in S.LEW congenic strain. (DOCX)

S24 Fig Secondary structure of *Rffl-lnc1* transcript 1 in 19bp knock-in targeted rescue model. (DOCX)

S25 Fig Secondary structure of *Rffl-lnc1* transcript 2 in 19bp knock-in targeted rescue model. (DOCX)

S26 Fig Secondary structure of *Rffl-lnc1* transcript 3 in 19bp knock-in targeted rescue model. (DOCX)

S27 Fig Secondary structure of *Rffl-lnc1* transcript 4 in 19bp knock-in targeted rescue model. (DOCX)

S1 Table Echocardiographic measurements in the S.LEW congenic strain and targeted rescue model. RWT, relative wall thickness; MPI, myocardial performance index; FS, fractional shortening; FS/MPI, functional index; SV, stroke volume; CO, cardiac output; CI, cardiac index; FSA, fractional shortening area. Experimental rats were maintained on low-salt diet after weaning and echocardiographic measurements were performed at about 14 weeks of age. All values are expressed as mean ± SEM. n = 8 rats/group.
(DOCX)

S2 Table Echocardiographic measurements in Dahl S rats and targeted rescue model. RWT, relative wall thickness; MPI, myocardial performance index; FS, fractional shortening; FS/MPI, functional index; SV, stroke volume; CO, cardiac output; CI, cardiac index; FSA, fractional shortening area. Experimental rats were maintained on low-salt diet after weaning and echocardiographic measurements were performed at about 11 weeks of age. All values are expressed as mean ± SEM. n = 8 rats/group.
(DOCX)
