# Supplementary material for: Positional cloning of quantitative trait nucleotides for blood pressure and cardiac QT-interval by targeted CRISPR/Cas9 editing of a novel long non-coding RNA
Source: PLoS Genet. 2017 Aug 21;13(8):e1006961. doi: 10.1371/journal.pgen.1006961 (PMC5578691; doi:10.1371/journal.pgen.1006961)
Supplement: S1 Fig — Rat C6 cells were transfected with gRNA and Cas9 encoding plasmids. Three days post-transfection, genomic DNA was harvested and used as template for the mismatch detection assay using the T7E1 enzyme. The uncleaved (508bp) amplicon and cleaved products (341bp and 167bp) are indicated. Cleavage products indicate active cleavage by rRffl.g4 with Cas9. (DOCX) [file pgen.1006961.s001.docx]

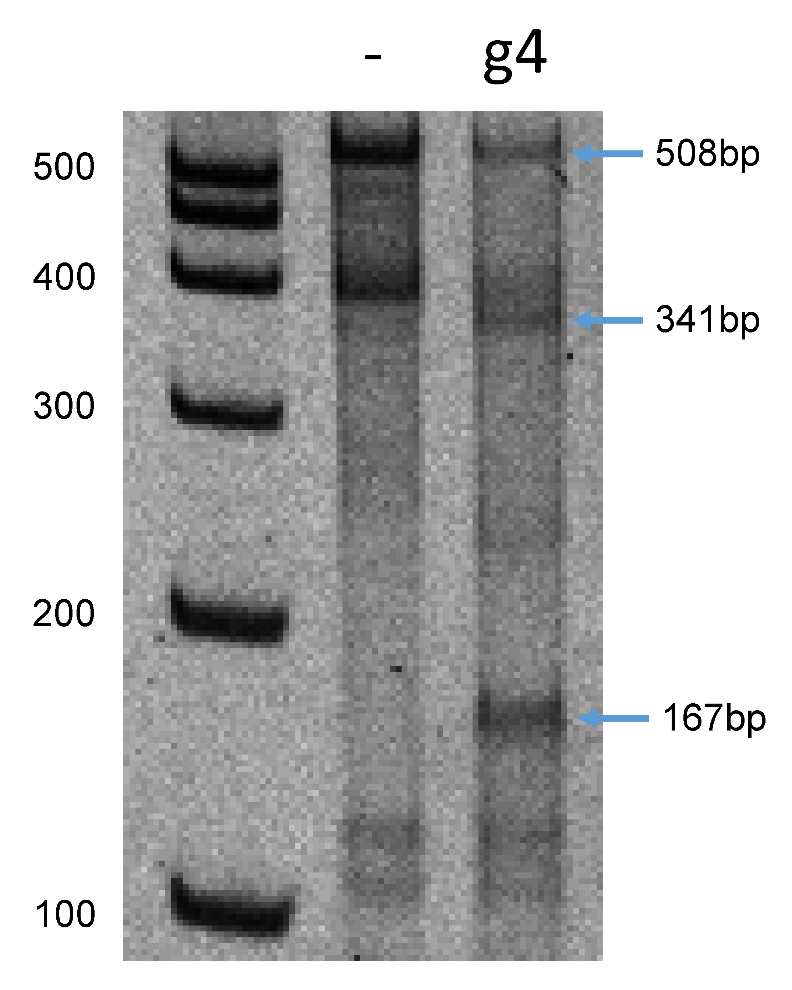


**Fig S1. In vitro validation of r*Rffl*.g4 using mismatch detection assay confirming cutting activity.** Rat C6 cells were transfected with gRNA and Cas9 encoding plasmids. Three days post-transfection, genomic DNA was harvested and used as template for the mismatch detection assay using the T7E1 enzyme. The uncleaved (508bp) amplicon and cleaved products (341bp and 167bp) are indicated. Cleavage products indicate active cleavage by r*Rffl*.g4 with Cas9.
