# Supplementary material for: Positional cloning of quantitative trait nucleotides for blood pressure and cardiac QT-interval by targeted CRISPR/Cas9 editing of a novel long non-coding RNA
Source: PLoS Genet. 2017 Aug 21;13(8):e1006961. doi: 10.1371/journal.pgen.1006961 (PMC5578691; doi:10.1371/journal.pgen.1006961)
Supplement: S2 Fig — (A) Rffl-lnc1 disruption model 1 versus S. (B) Rffl-lnc1 disruption model 2 versus S. (C) Rffl-lnc1 disruption model 3 versus S. (D) Rffl-lnc1 disruption model 4 versus S. Hearts were collected within one week after blood pressure data (Fig 2) was collected. All values are expressed as mean ± SEM. n = 7–9 rats/group. (DOCX) [file pgen.1006961.s002.docx]

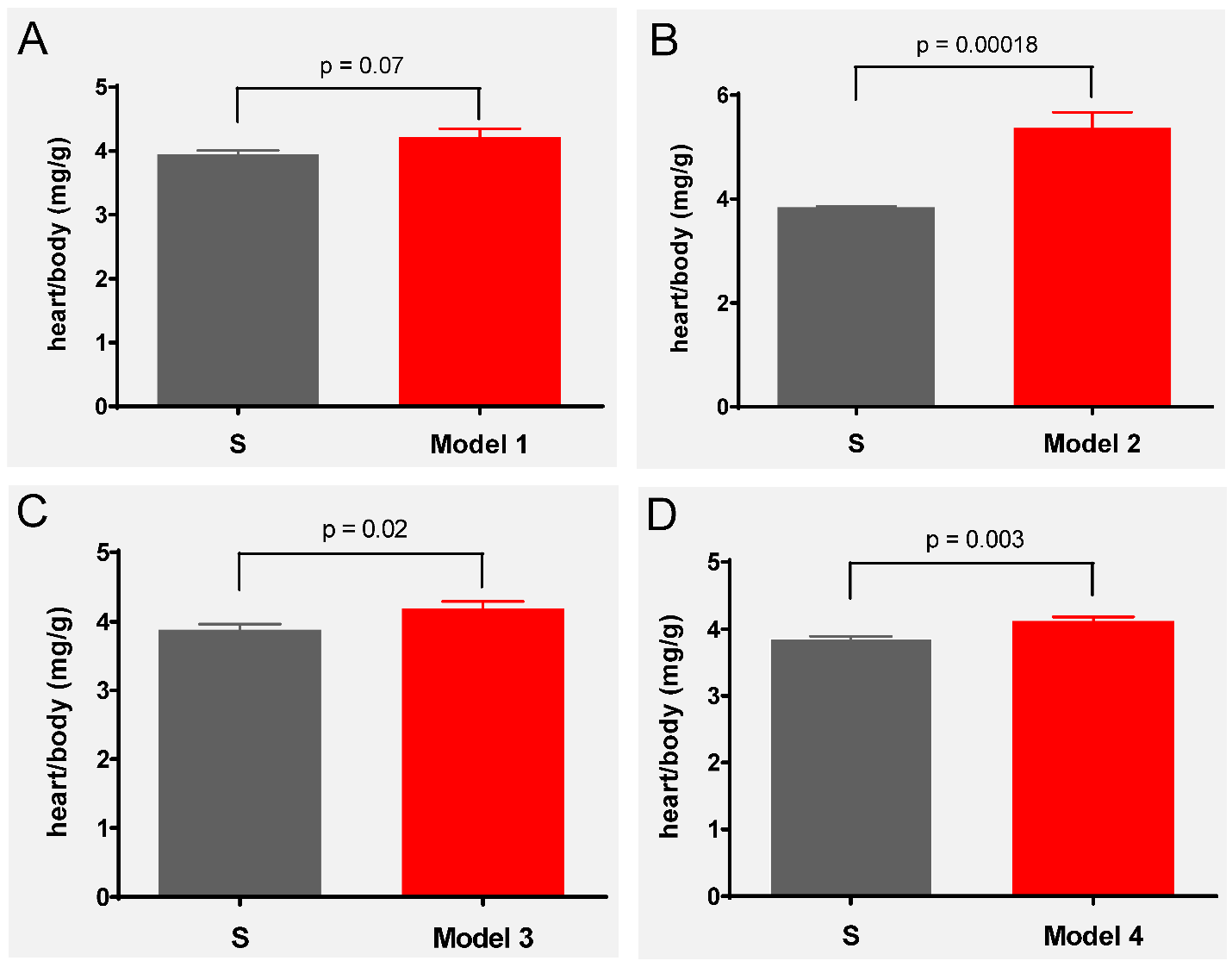


**Fig S2. The heart weight/body weight ratios were higher in *Rffl-lnc1* disruption models compared with S rats.** (A) *Rffl-lnc1* disruption model 1 versus S. (B) *Rffl-lnc1* disruption model 2 versus S. (C) *Rffl-lnc1* disruption model 3 versus S. (D) *Rffl-lnc1* disruption model 4 versus S. Hearts were collected within one week after blood pressure data (Fig 2) was collected. All values are expressed as mean ± SEM. n = 7-9 rats/group.
