# Supplementary figures and images for: Positional cloning of quantitative trait nucleotides for blood pressure and cardiac QT-interval by targeted CRISPR/Cas9 editing of a novel long non-coding RNA
Source: PLoS Genet. 2017 Aug 21;13(8):e1006961. doi: 10.1371/journal.pgen.1006961 (PMC5578691; doi:10.1371/journal.pgen.1006961)

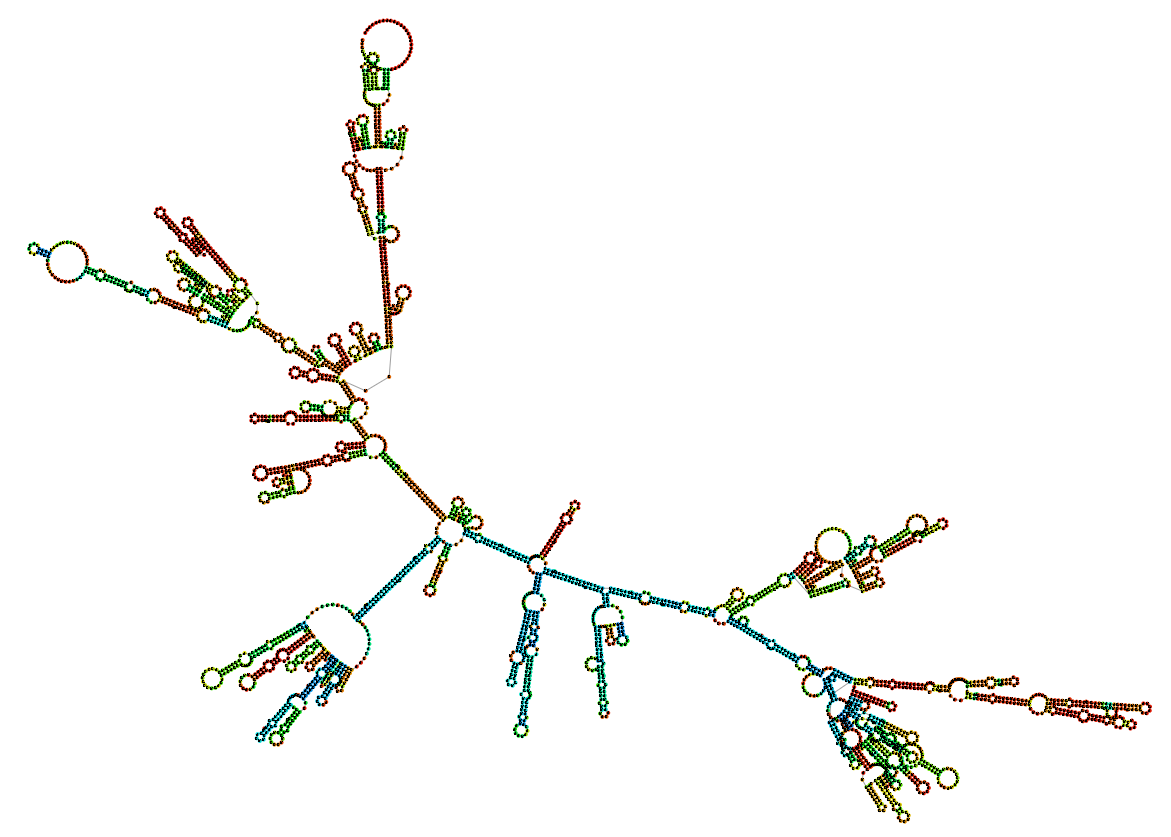


**Fig S3. Secondary structure of *Rffl-lnc1* transcript 1 in Dahl S rat.**

Supplement: S3 Fig — (DOCX) [file pgen.1006961.s003.docx]

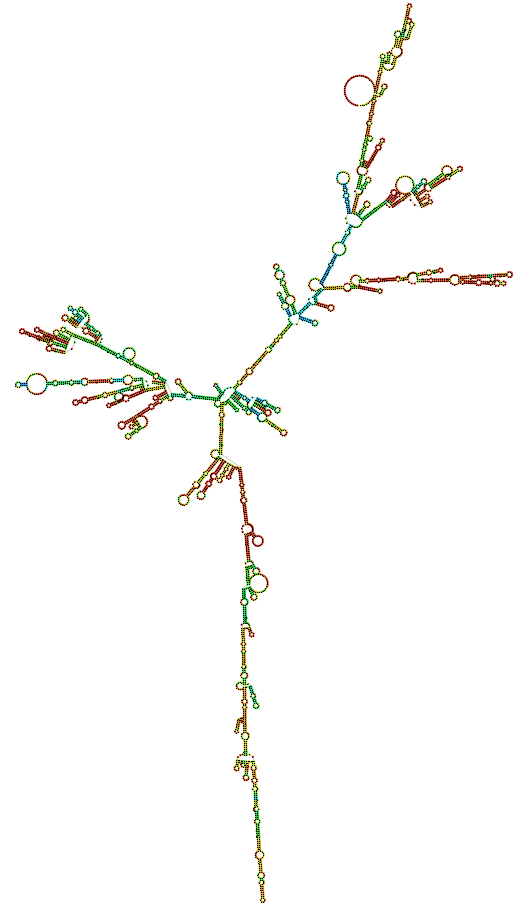


**Fig S4. Secondary structure of *Rffl-lnc1* transcript 2 in Dahl S rat.**

Supplement: S4 Fig — (DOCX) [file pgen.1006961.s004.docx]

*
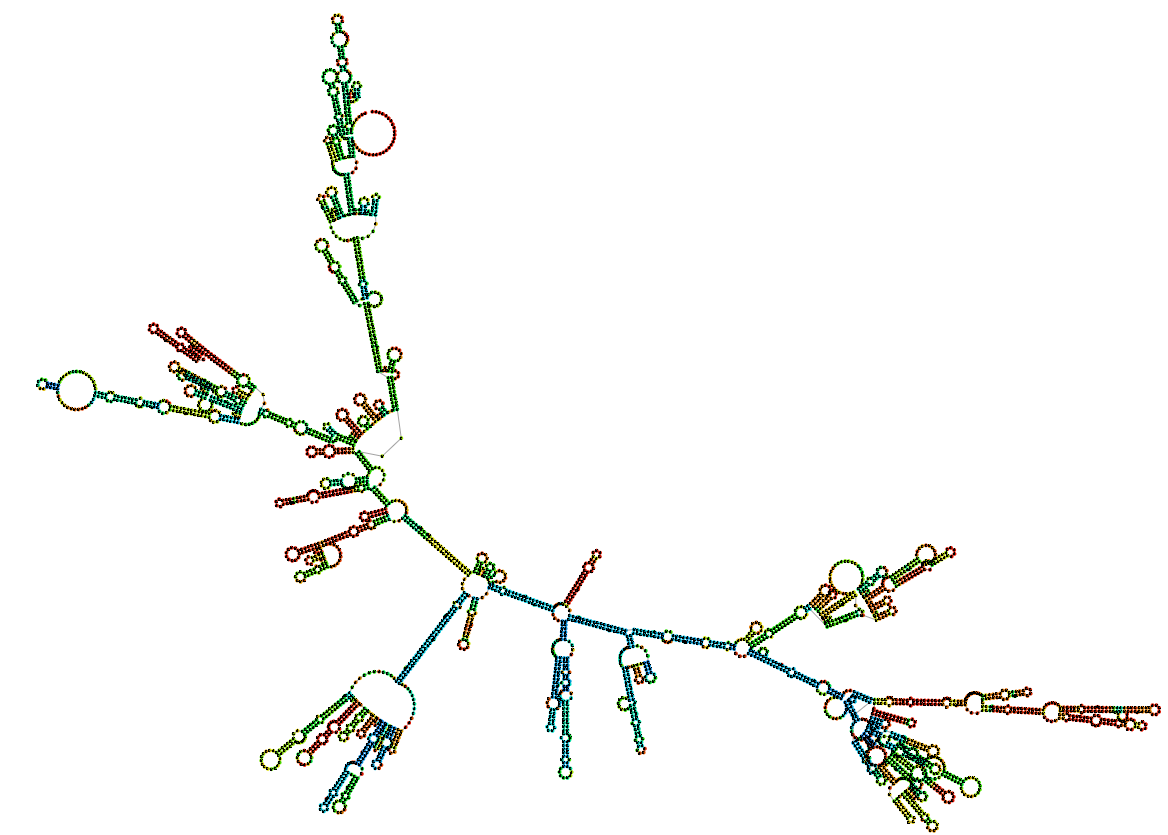
*

**Fig S5. Secondary structure of *Rffl-lnc1* transcript 3 in Dahl S rat.**

Supplement: S5 Fig — (DOCX) [file pgen.1006961.s005.docx]

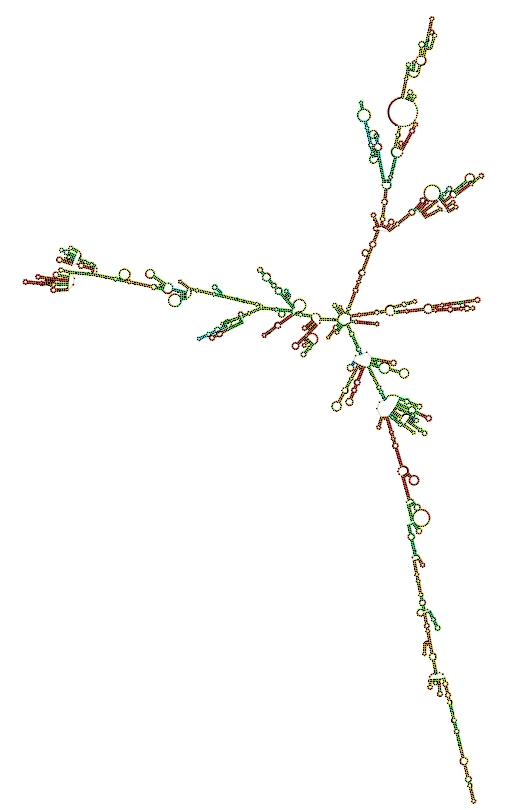


**Fig S6. Secondary structure of *Rffl-lnc1* transcript 4 in Dahl S rat.**

Supplement: S6 Fig — (DOCX) [file pgen.1006961.s006.docx]

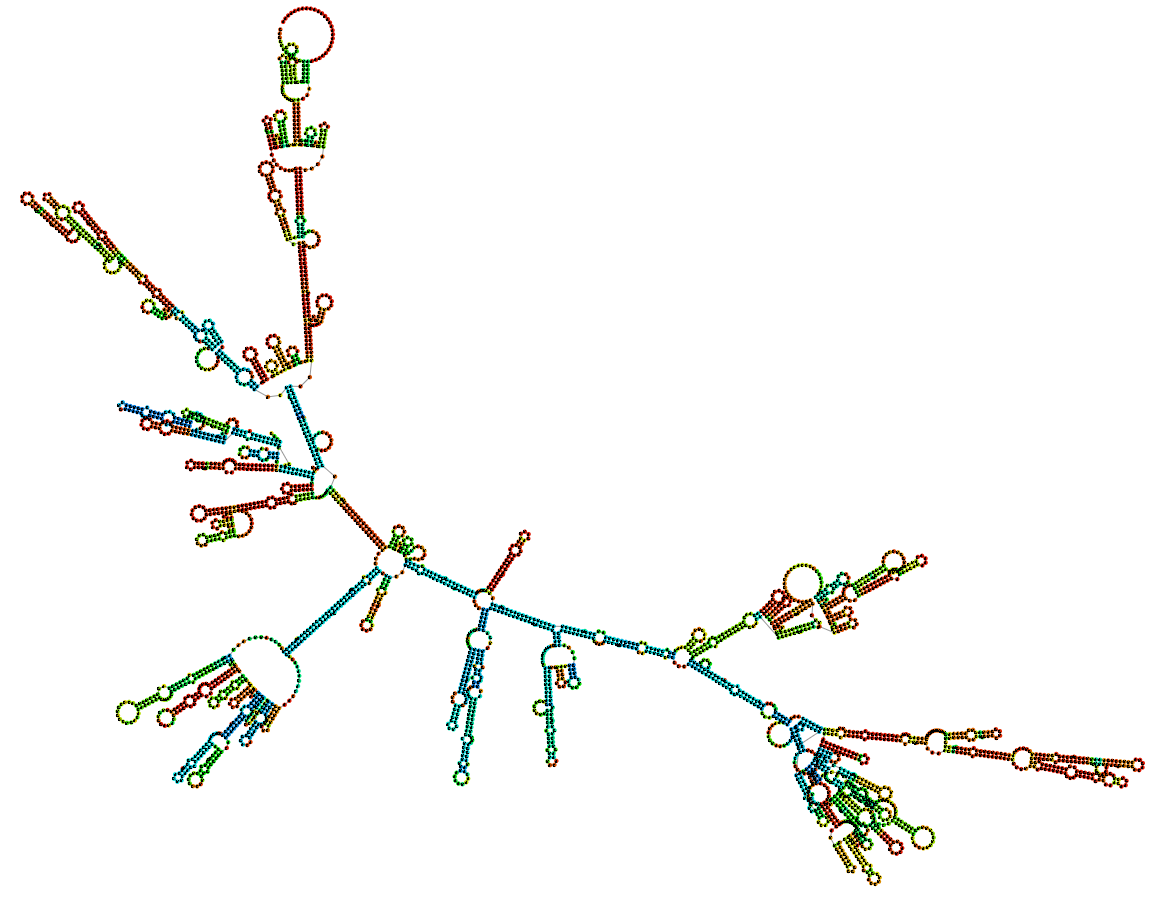


**Fig S7. Secondary structure of *Rffl-lnc1* transcript 1 in *Rffl-lnc1* disruption model 1.**

Supplement: S7 Fig — (DOCX) [file pgen.1006961.s007.docx]

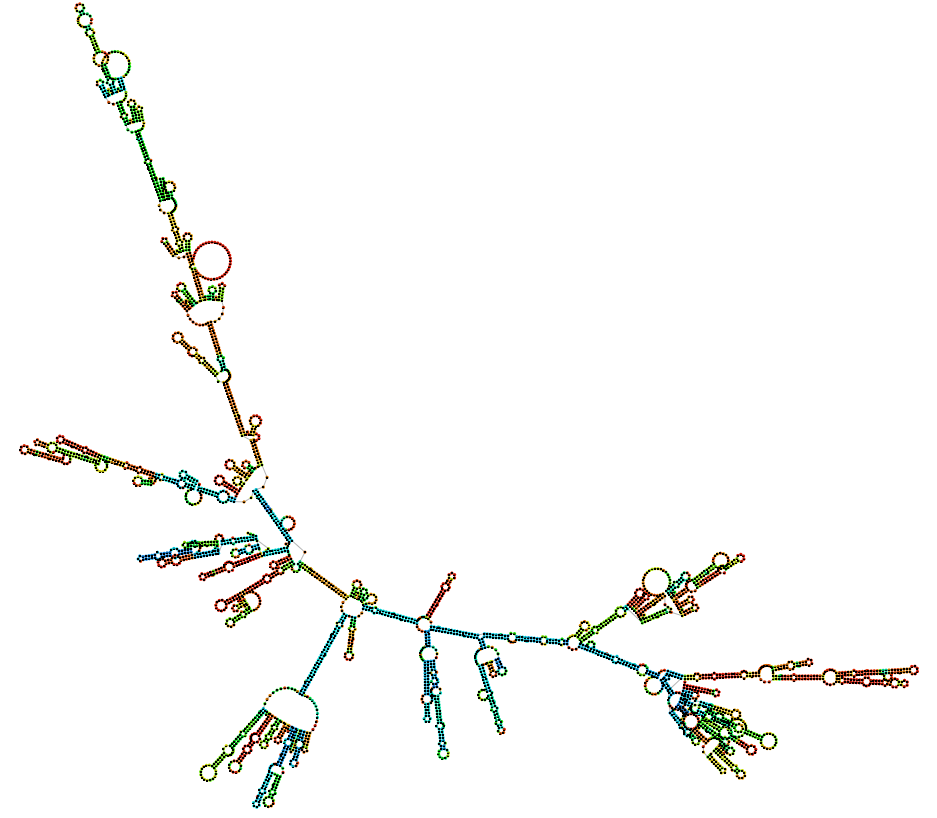


**Fig S8. Secondary structure of *Rffl-lnc1* transcript 2 in *Rffl-lnc1* disruption model 1.**

Supplement: S8 Fig — (DOCX) [file pgen.1006961.s008.docx]

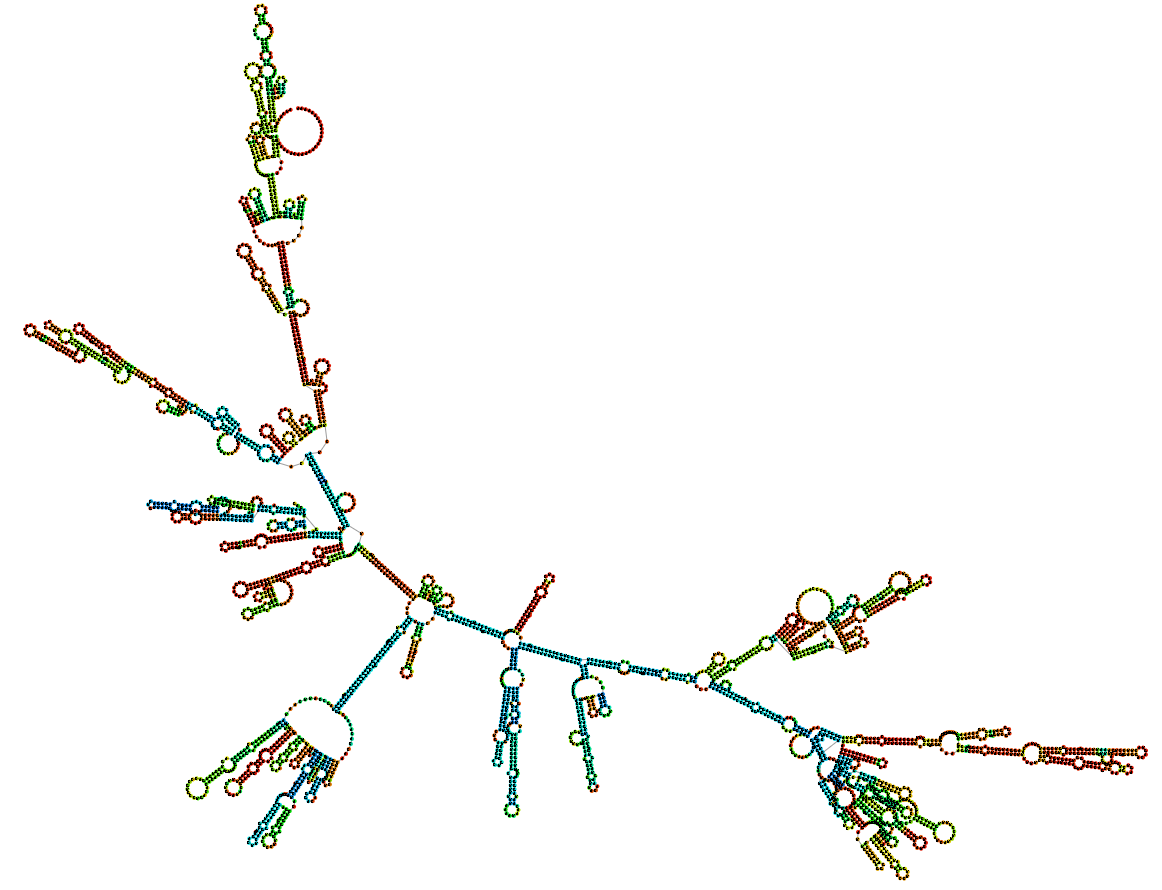


**Fig S9. Secondary structure of *Rffl-lnc1* transcript 3 in *Rffl-lnc1* disruption model 1.**

Supplement: S9 Fig — (DOCX) [file pgen.1006961.s009.docx]

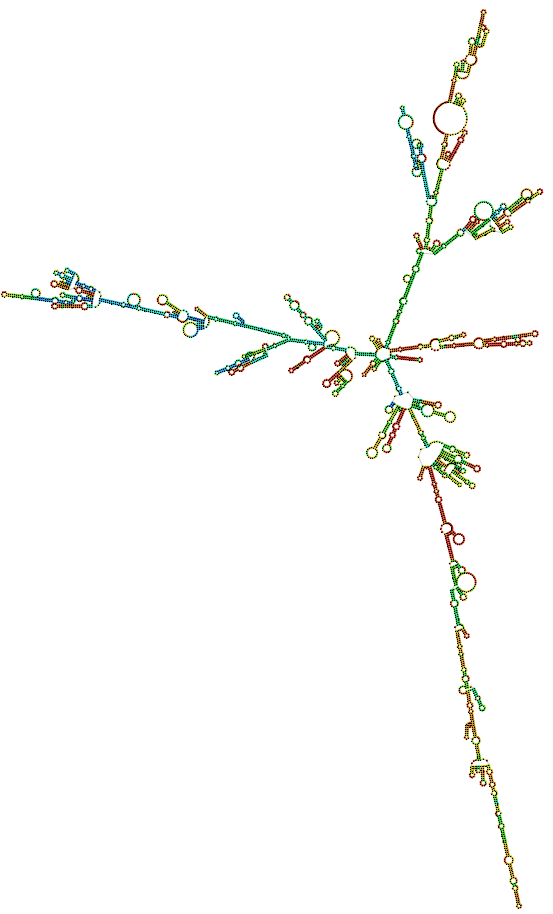


**Fig S10. Secondary structure of *Rffl-lnc1* transcript 4 in *Rffl-lnc1* disruption model 1.**

Supplement: S10 Fig — (DOCX) [file pgen.1006961.s010.docx]

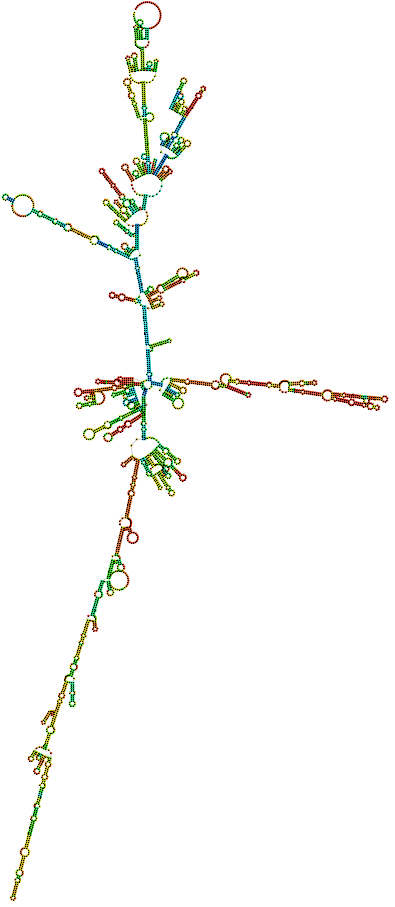


**Fig S11. Secondary structure of *Rffl-lnc1* transcript 1 in *Rffl-lnc1* disruption model 2.**

Supplement: S11 Fig — (DOCX) [file pgen.1006961.s011.docx]

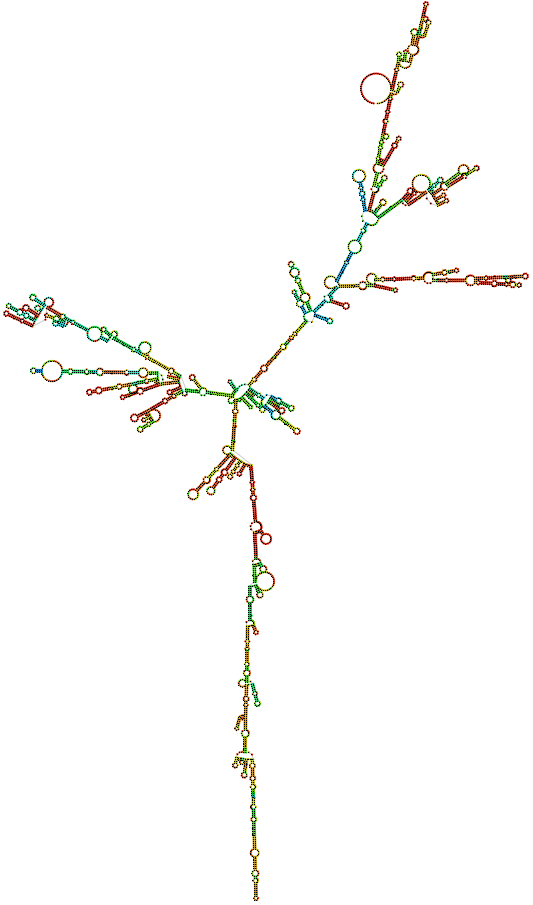


**Fig S12. Secondary structure of *Rffl-lnc1* transcript 2 in *Rffl-lnc1* disruption model 2.**

Supplement: S12 Fig — (DOCX) [file pgen.1006961.s012.docx]

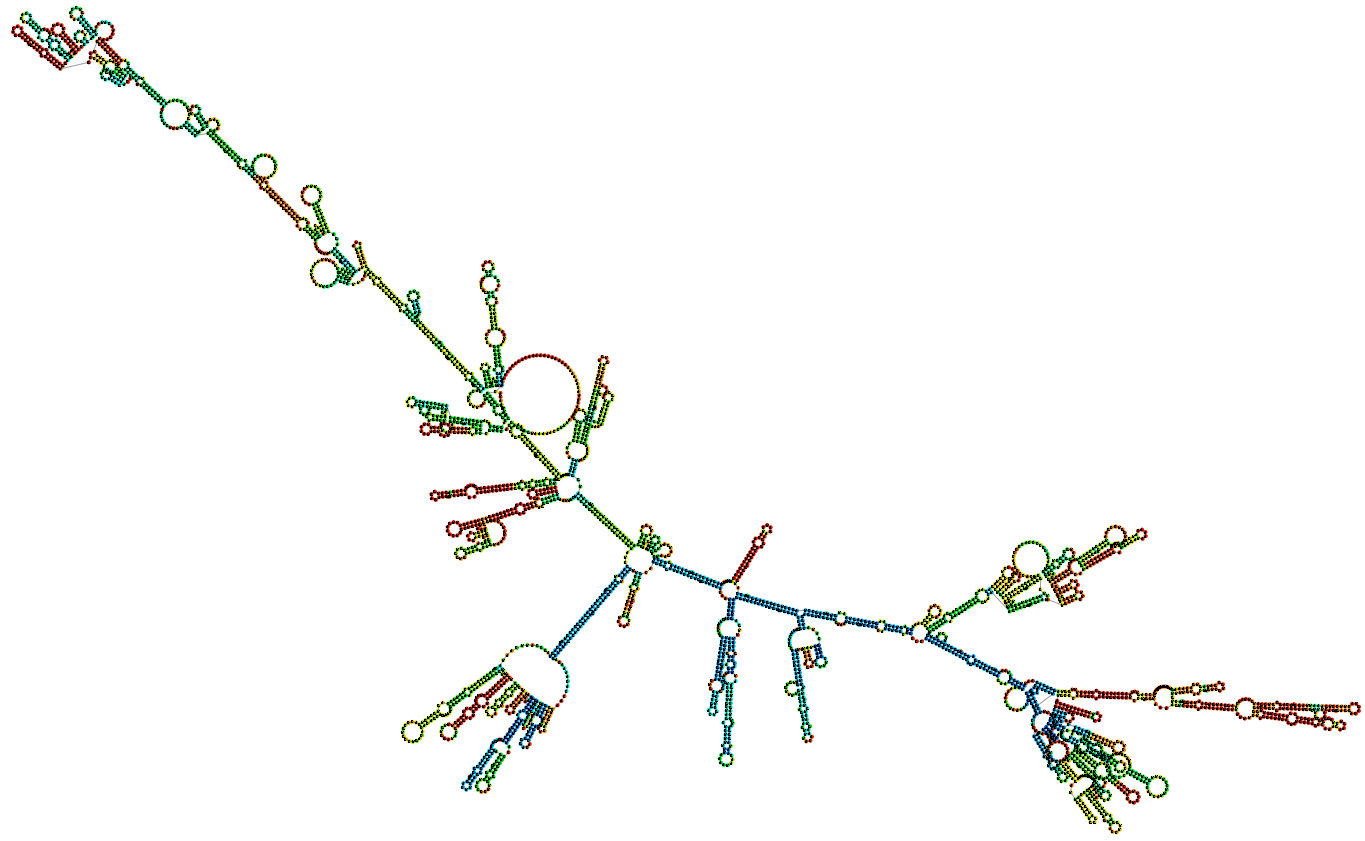


**Fig S13. Secondary structure of *Rffl-lnc1* transcript 3 in *Rffl-lnc1* disruption model 2.**

Supplement: S13 Fig — (DOCX) [file pgen.1006961.s013.docx]

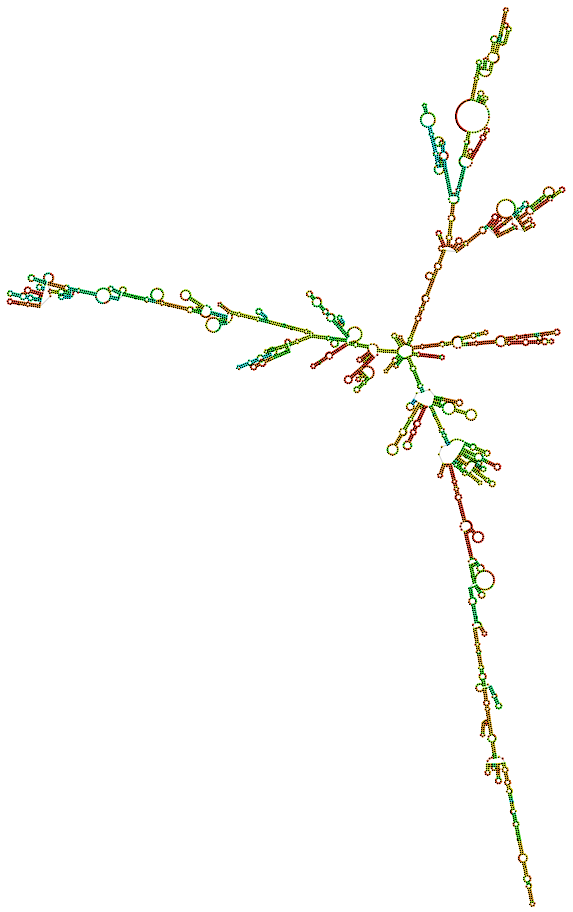


**Fig S14. Secondary structure of *Rffl-lnc1* transcript 4 in *Rffl-lnc1* disruption model 2.**

Supplement: S14 Fig — (DOCX) [file pgen.1006961.s014.docx]

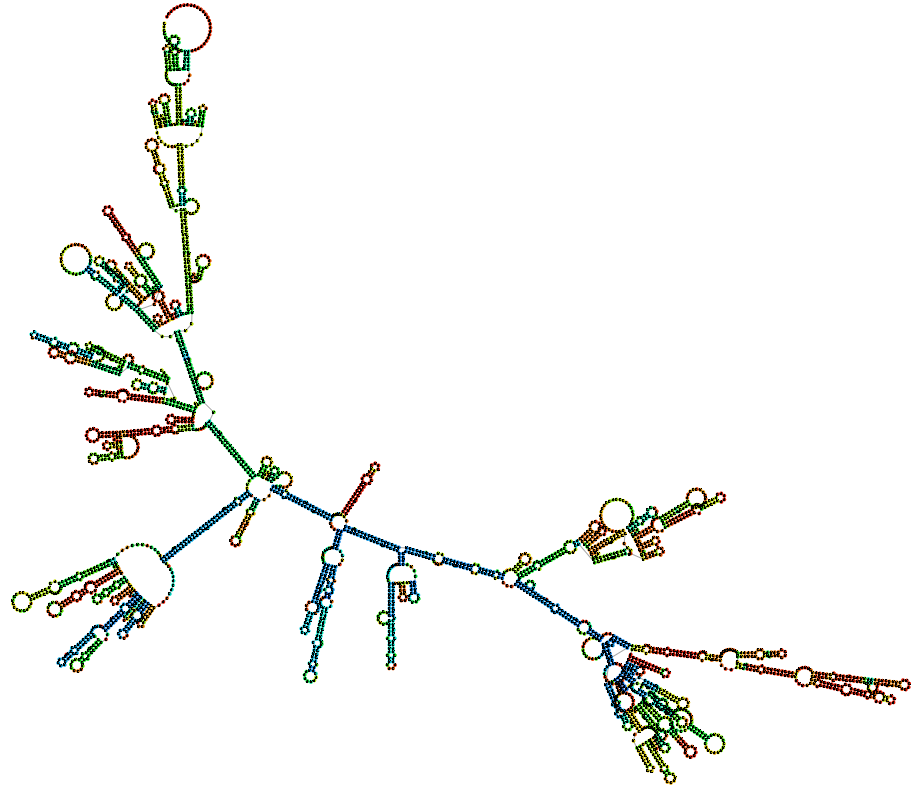


**Fig S15. Secondary structure of *Rffl-lnc1* transcript 1 in *Rffl-lnc1* disruption model 3.**

Supplement: S15 Fig — (DOCX) [file pgen.1006961.s015.docx]

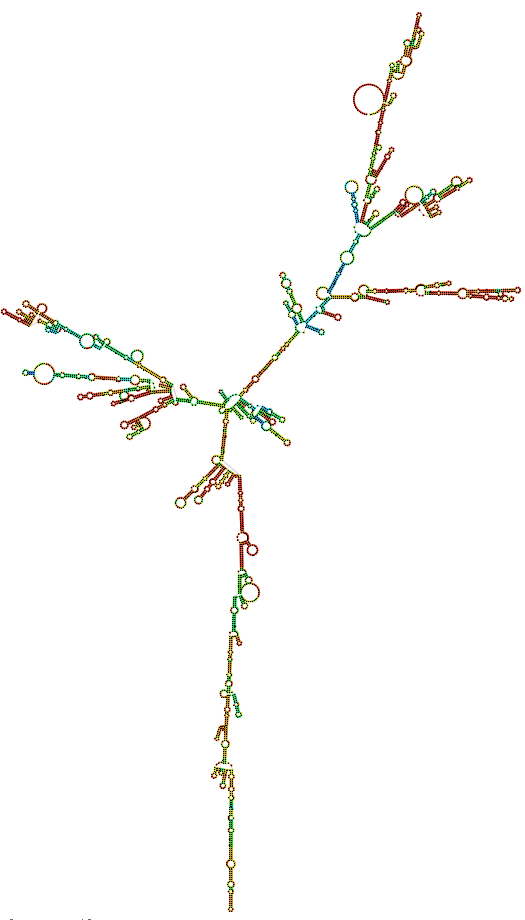


**Fig S16. Secondary structure of *Rffl-lnc1* transcript 2 in *Rffl-lnc1* disruption model 3.**

Supplement: S16 Fig — (DOCX) [file pgen.1006961.s016.docx]

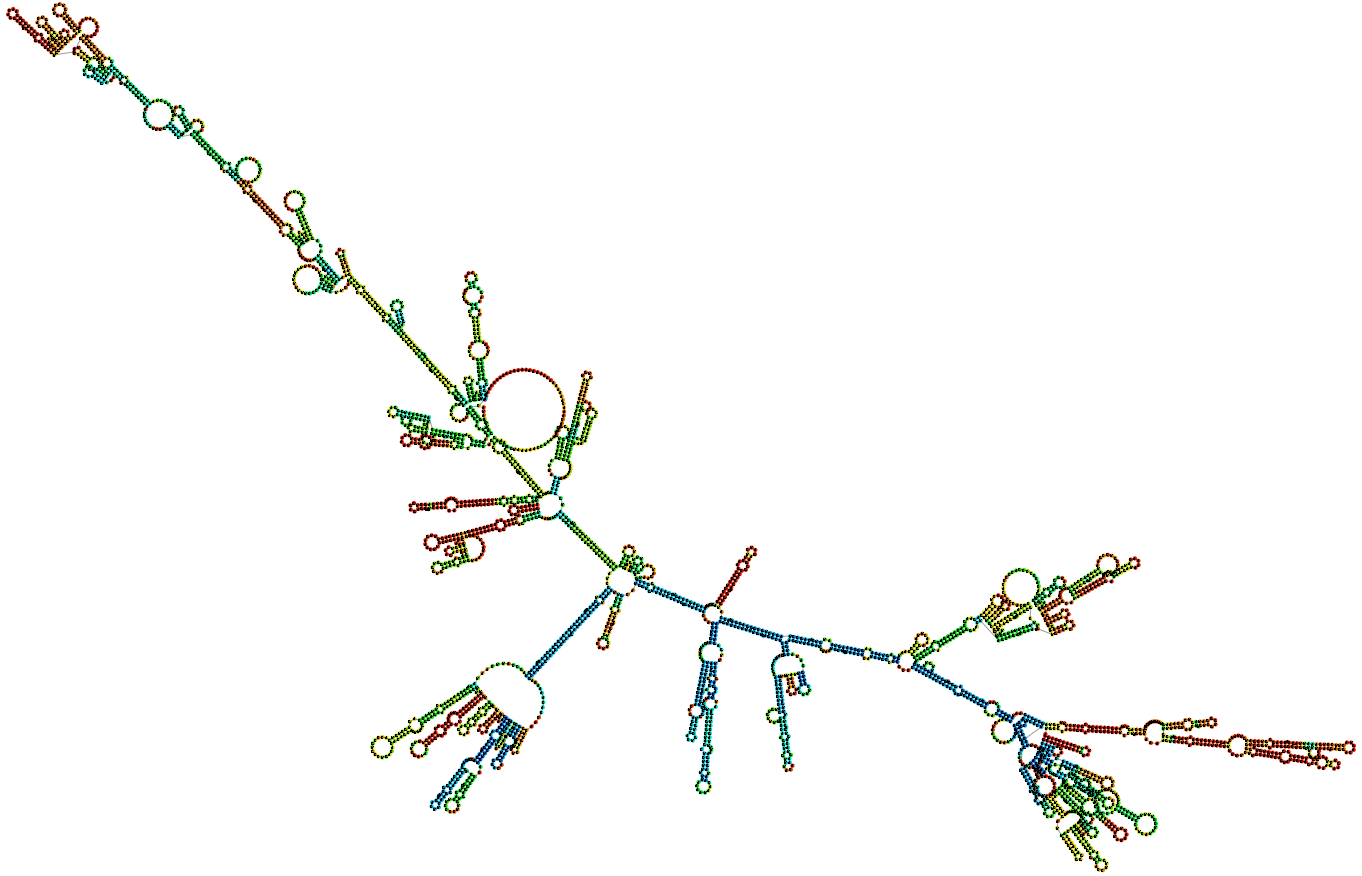


**Fig S17. Secondary structure of *Rffl-lnc1* transcript 3 in *Rffl-lnc1* disruption model 3.**

Supplement: S17 Fig — (DOCX) [file pgen.1006961.s017.docx]

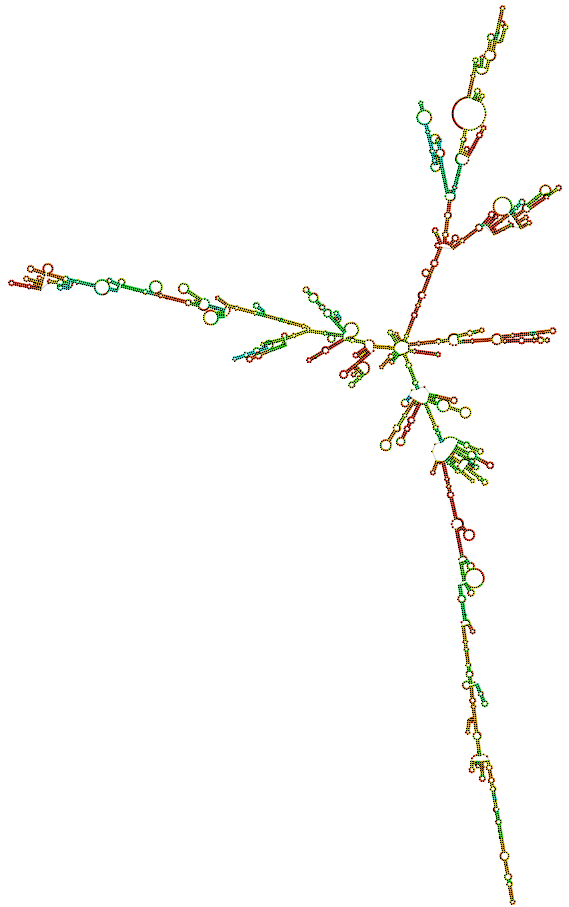


**Fig S18. Secondary structure of *Rffl-lnc1* transcript 4 in *Rffl-lnc1* disruption model 3.**

Supplement: S18 Fig — (DOCX) [file pgen.1006961.s018.docx]

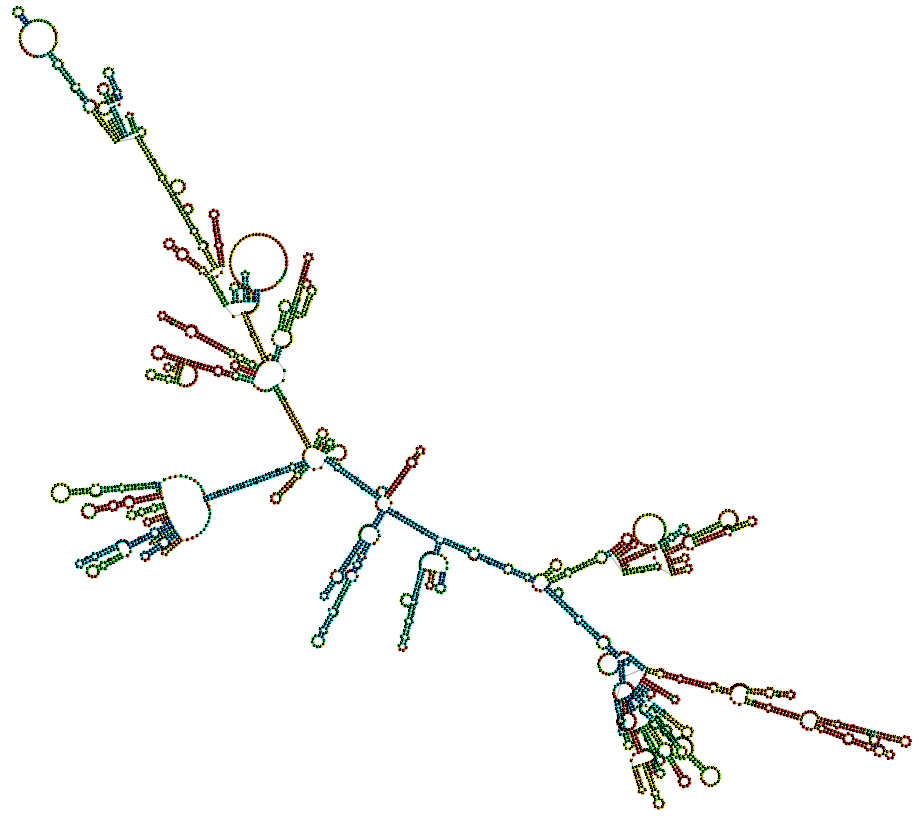


**Fig S19. Secondary structure of *Rffl-lnc1* transcript in *Rffl-lnc1* disruption model 4.**

Supplement: S19 Fig — (DOCX) [file pgen.1006961.s019.docx]

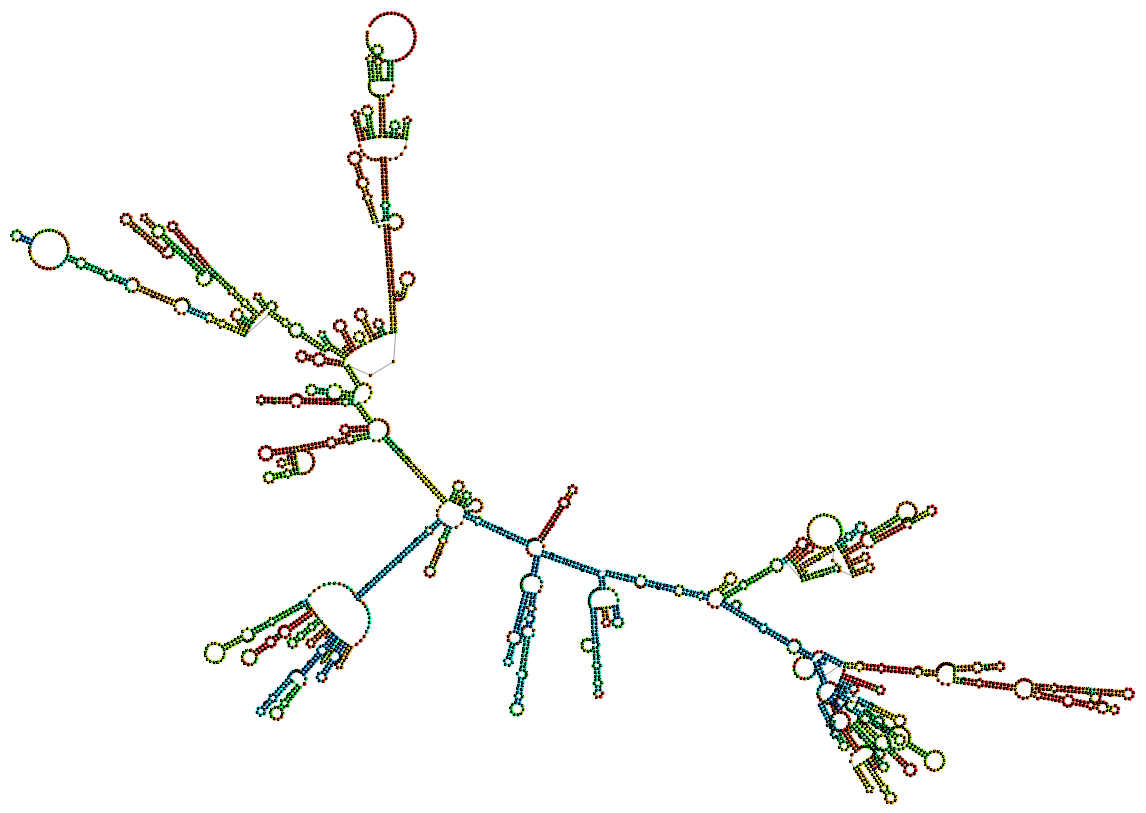


**Fig S20. Secondary structure of *Rffl-lnc1* transcript 1 in S.LEW congenic strain.**

Supplement: S20 Fig — (DOCX) [file pgen.1006961.s020.docx]

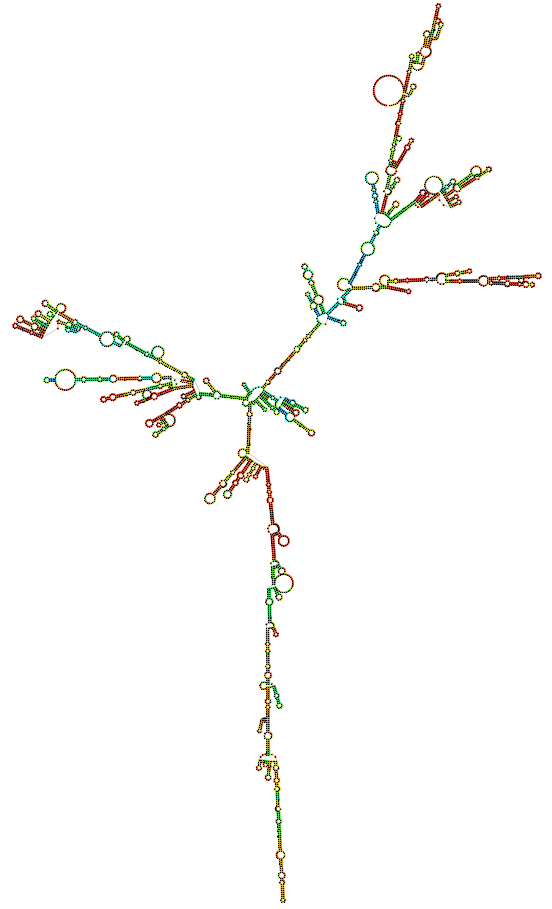


**Fig S21. Secondary structure of *Rffl-lnc1* transcript 2 in S.LEW congenic strain.**

Supplement: S21 Fig — (DOCX) [file pgen.1006961.s021.docx]

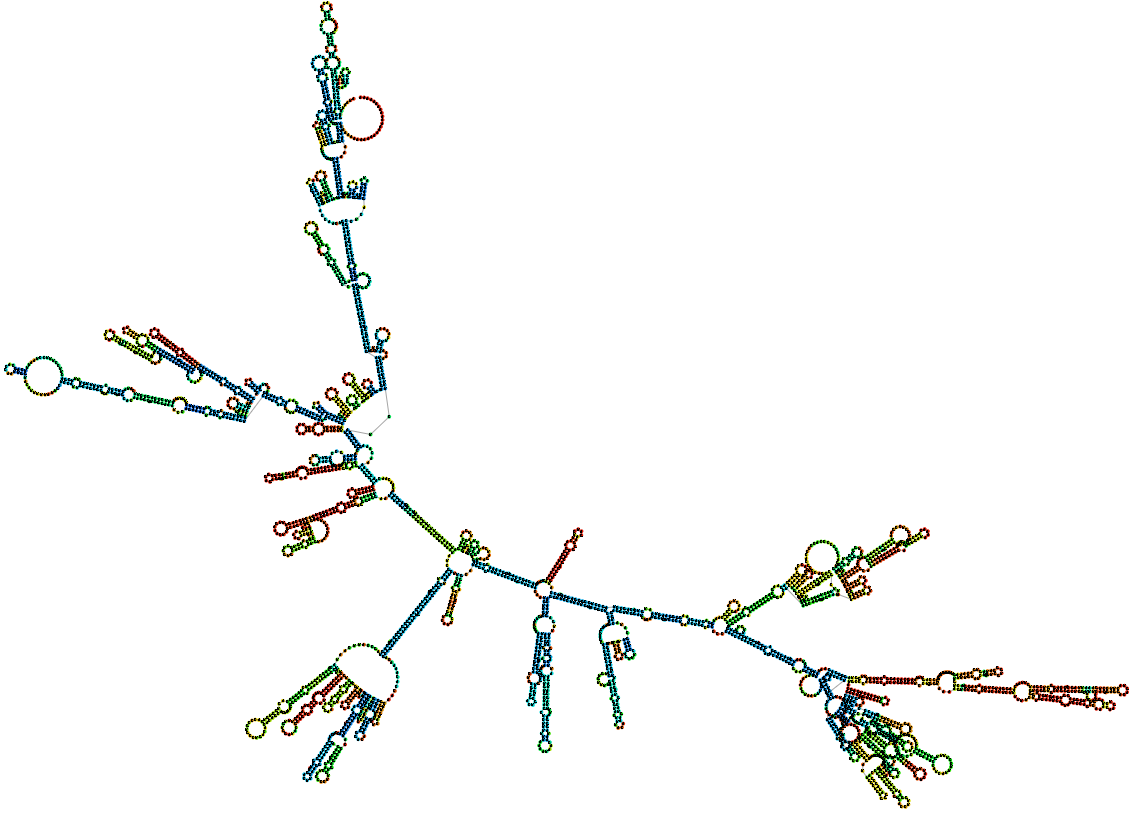


**Fig S22. Secondary structure of *Rffl-lnc1* transcript 3 in S.LEW congenic strain.**

Supplement: S22 Fig — (DOCX) [file pgen.1006961.s022.docx]

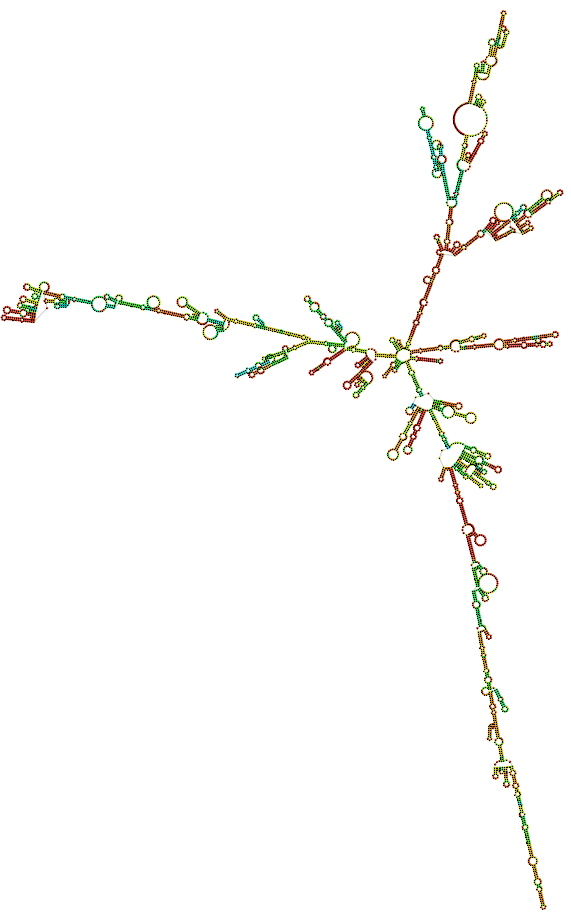


**Fig S23. Secondary structure of *Rffl-lnc1* transcript 4 in S.LEW congenic strain.**

Supplement: S23 Fig — (DOCX) [file pgen.1006961.s023.docx]

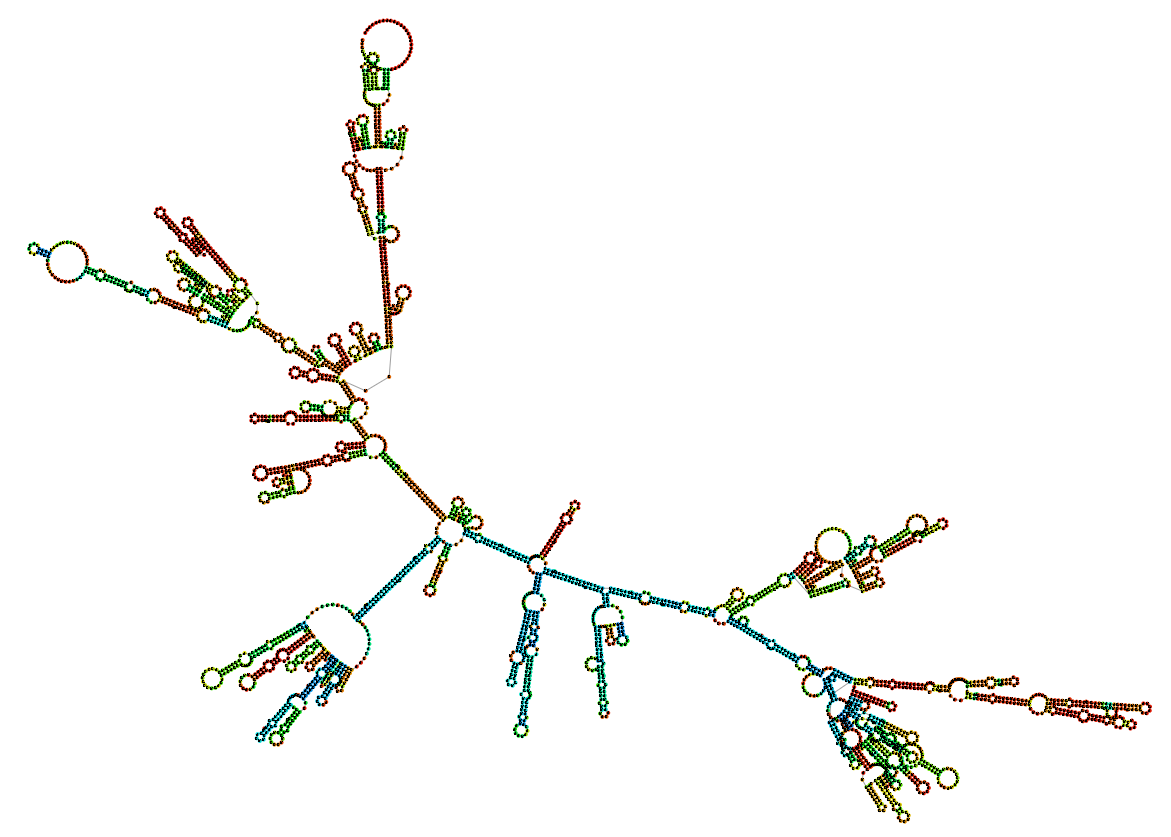


**Fig S24. Secondary structure of *Rffl-lnc1* transcript 1 in 19bp knock-in targeted rescue model.**

Supplement: S24 Fig — (DOCX) [file pgen.1006961.s024.docx]

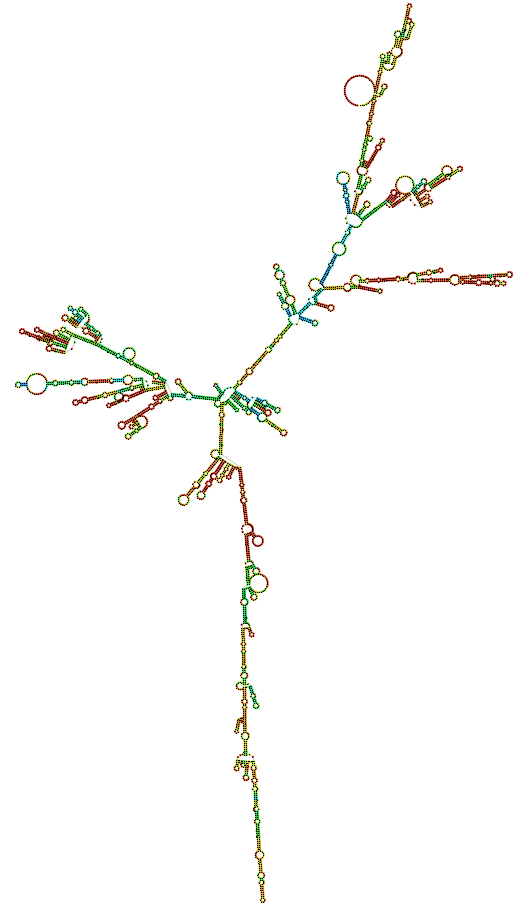


**Fig S25. Secondary structure of *Rffl-lnc1* transcript 2 in 19bp knock-in targeted rescue model.**

Supplement: S25 Fig — (DOCX) [file pgen.1006961.s025.docx]

*
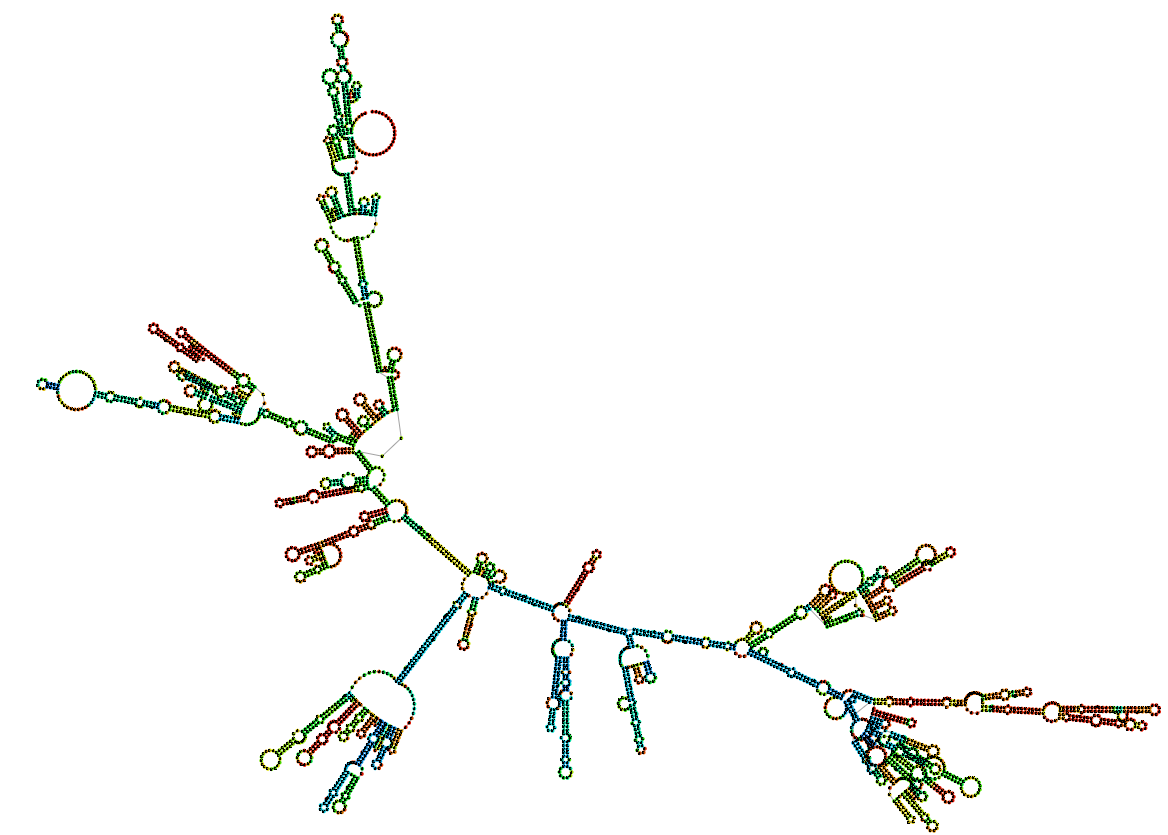
*

**Fig S26. Secondary structure of *Rffl-lnc1* transcript 3 in 19bp knock-in targeted rescue model.**

Supplement: S26 Fig — (DOCX) [file pgen.1006961.s026.docx]

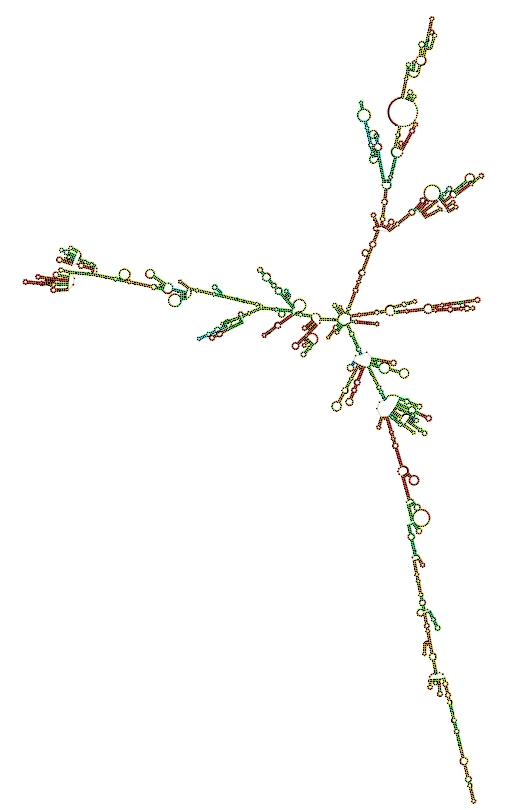


**Fig S27. Secondary structure of *Rffl-lnc1* transcript 4 in 19bp knock-in targeted rescue model.**

Supplement: S27 Fig — (DOCX) [file pgen.1006961.s027.docx]
