# Supplementary material for: Positional cloning of quantitative trait nucleotides for blood pressure and cardiac QT-interval by targeted CRISPR/Cas9 editing of a novel long non-coding RNA
Source: PLoS Genet. 2017 Aug 21;13(8):e1006961. doi: 10.1371/journal.pgen.1006961 (PMC5578691; doi:10.1371/journal.pgen.1006961)
Supplement: S1 Table — RWT, relative wall thickness; MPI, myocardial performance index; FS, fractional shortening; FS/MPI, functional index; SV, stroke volume; CO, cardiac output; CI, cardiac index; FSA, fractional shortening area. Experimental rats were maintained on low-salt diet after weaning and echocardiographic measurements were performed at about 14 weeks of age. All values are expressed as mean ± SEM. n = 8 rats/group. (DOCX) [file pgen.1006961.s028.docx]

**Table S1. Echocardiographic measurements in the S.LEW congenic strain and targeted rescue model**

| Parameter | S.LEW congenic | Targeted Rescue | p-value |
| --- | --- | --- | --- |
| RWT | 0.59 ± 0.03 | 0.54 ± 0.03 | 0.34 |
| MPI | 0.37 ± 0.03 | 0.28 ± 0.02 | 0.02 |
| FS/MPI | 1.37 ± 0.08 | 1.92 ± 0.24 | 0.045 |
| SV (ml) | 0.10 ± 0.01 | 0.12 ± 0.01 | 0.34 |
| CO (ml/min) | 45 ± 3 | 49 ± 6 | 0.56 |
| CI (ml/min/kg) | 125 ± 7 | 129 ± 15 | 0.83 |
| FS | 0.50 ± 0.01 | 0.51 ± 0.02 | 0.73 |
| FSA | 0.50 ± 0.02 | 0.53 ± 0.03 | 0.46 |

RWT, relative wall thickness; MPI, myocardial performance index; FS, fractional shortening; FS/MPI, functional index; SV, stroke volume; CO, cardiac output; CI, cardiac index; FSA, fractional shortening area.

Experimental rats were maintained on low-salt diet after weaning and echocardiographic measurements were performed at about 14 weeks of age. All values are expressed as mean ± SEM. n = 8 rats/group.
