# Supplementary material for: Positional cloning of quantitative trait nucleotides for blood pressure and cardiac QT-interval by targeted CRISPR/Cas9 editing of a novel long non-coding RNA
Source: PLoS Genet. 2017 Aug 21;13(8):e1006961. doi: 10.1371/journal.pgen.1006961 (PMC5578691; doi:10.1371/journal.pgen.1006961)
Supplement: S2 Table — RWT, relative wall thickness; MPI, myocardial performance index; FS, fractional shortening; FS/MPI, functional index; SV, stroke volume; CO, cardiac output; CI, cardiac index; FSA, fractional shortening area. Experimental rats were maintained on low-salt diet after weaning and echocardiographic measurements were performed at about 11 weeks of age. All values are expressed as mean ± SEM. n = 8 rats/group. (DOCX) [file pgen.1006961.s029.docx]

**Table S2. Echocardiographic measurements in Dahl S rats and targeted rescue model**

| Parameter | S | Targeted Rescue | p-value |
| --- | --- | --- | --- |
| RWT | 0.54 ± 0.03 | 0.57 ± 0.05 | 0.64 |
| MPI | 0.27 ± 0.02 | 0.32 ± 0.02 | 0.19 |
| FS/MPI | 2.10 ± 0.18 | 1.62 ± 0.21 | 0.09 |
| SV (ml) | 0.14 ± 0.01 | 0.12 ± 0.01 | 0.15 |
| CO (ml/min) | 63 ± 4 | 55 ± 5 | 0.21 |
| CI (ml/min/kg) | 189 ± 10 | 169 ± 15 | 0.28 |
| FS | 0.55 ± 0.02 | 0.48 ± 0.03 | 0.07 |
| FSA | 0.58 ± 0.01 | 0.54 ± 0.02 | 0.10 |

RWT, relative wall thickness; MPI, myocardial performance index; FS, fractional shortening; FS/MPI, functional index; SV, stroke volume; CO, cardiac output; CI, cardiac index; FSA, fractional shortening area.

Experimental rats were maintained on low-salt diet after weaning and echocardiographic measurements were performed at about 11 weeks of age. All values are expressed as mean ± SEM. n = 8 rats/group.
